# *GAA* variants and phenotypes among 1,079 patients with Pompe disease: Data from the Pompe Registry

**DOI:** 10.1002/humu.23878

**Published:** 2019-08-07

**Authors:** Arnold J. J. Reuser, Ans T. van der Ploeg, Yin‐Hsiu Chien, Juan Llerena, Mary‐Alice Abbott, Paula R. Clemens, Virginia E. Kimonis, Nancy Leslie, Sonia S. Maruti, Bernd‐Jan Sanson, Roberto Araujo, Magali Periquet, Antonio Toscano, Priya S. Kishnani, on behalf of the Pompe Registry Sites

**Affiliations:** ^1^ Department of Clinical Genetics Erasmus MC University Medical Center Rotterdam The Netherlands; ^2^ Center for Lysosomal and Metabolic Diseases Erasmus MC University Medical Center Rotterdam The Netherlands; ^3^ Department of Medical Genetics National Taiwan University Hospital Taipei Taiwan; ^4^ Departamento de Genética Médica Instituto Fernandes Figueira (FIOCRUZ) Rio de Janeiro RJ Brazil; ^5^ Department of Pediatrics Baystate Medical Center Springfield Massachusetts; ^6^ Department of Neurology and Department of Veterans Affairs Medical Center University of Pittsburgh Pittsburgh Pennsylvania; ^7^ Division of Genetics and Genomic Medicine, Department of Pediatrics, School of Medicine University of California Irvine California; ^8^ Division of Human Genetics Cincinnati Children's Hospital Medical Center Cincinnati Ohio; ^9^ Sanofi Genzyme Cambridge Massachusetts; ^10^ Sanofi Genzyme Amsterdam The Netherlands; ^11^ Department of Clinical and Experimental Medicine, Reference Center for Rare Neuromuscular Disorders University of Messina Messina Italy; ^12^ Division of Medical Genetics, Department of Pediatrics Duke University Medical Center Durham North Carolina

**Keywords:** acid α‐glucosidase, *GAA* genotypes, *GAA* variants, Pompe disease; Pompe Registry

## Abstract

Identification of variants in the acid α‐glucosidase (*GAA*) gene in Pompe disease provides valuable insights and systematic overviews are needed. We report on the number, nature, frequency, and geographic distribution of *GAA* sequence variants listed in the Pompe Registry, a long‐term, observational program and the largest global repository of Pompe disease data. Variant information was reviewed and compared with publicly available *GAA* databases/resources. Among 1,079 eligible patients, 2,075 *GAA* variants (80 unique novel) were reported. Variants were listed by groups representing Pompe disease phenotypes. Patients were classified as Group A: Symptom onset ≤ 12 months of age with cardiomyopathy; Group B: Symptom onset ≤ 12 years of age (includes patients with symptom onset ≤ 12 months of age without cardiomyopathy); or Group C: Symptom onset > 12 years of age. Likely impact of novel variants was predicted using bioinformatics algorithms. Variants were classified by pathogenicity using ACMG guidelines. Data reported from the Pompe Registry provide new information about the distribution of *GAA* variants globally and across the clinical spectrum, add to the number and diversity of *GAA* variants registered in public databases through published data sharing, provide a first indication of the severity of novel variants, and assist in diagnostic practice and outcome prediction.

## 1 INTRODUCTION

### 1.1 Overview of Pompe disease

Pompe disease, also known as acid maltase deficiency and glycogen storage disease type II (MIM# 232300), is a rare, progressive, autosomal recessive inheritance (HP:0000007) disorder caused by deficient acid α‐glucosidase (GAA), the lysosomal enzyme that breaks down glycogen. Resulting abnormal accumulation of glycogen in lysosomes leads to cellular dysfunction; progressive respiratory failure due to muscle weakness (HP:0002740), cardiac anomalies (HP:0001626), abnormal skeletal muscle morphology (HP:0004303), and damage to smooth muscles with related functional disabilities; significant morbidity; and premature age of death (HP:0011420) in many patients (Kishnani, Hwu et al., 2006; Reuser, Hirschhorn, & Kroos, 2018; van der Ploeg & Reuser, 2008; van der Ploeg et al., 2017). While all patients eventually exhibit progressive muscle weakness (HP:0003323), Pompe disease manifests as a broad clinical spectrum of phenotypes with considerable variation in age of symptom onset, presenting signs/symptoms, degree of severity, organ involvement, and rate of progression. Patients with the most severe form, classic infantile Pompe disease, have onset of symptoms ≤ 12 months of age, often within the first days to weeks of life, with progressive cardiomyopathy (HP:0001638), decreased respiratory function due to muscle weakness (HP:0002747), muscle hypotonia (HP:0001252), and death in infancy (between 1 and 2 years of age; HP:0001522; Kishnani, Hwu et al., 2006; Reuser et al., 2018; van den Hout et al., 2003). Cardiomyopathy is a cardinal feature of classic infantile Pompe disease. In other patients, symptoms, including progressive skeletal muscle weakness (HP:0040290) affecting mobility and leading to respiratory failure (HP:0002093), evolve as a continuum of disease, presenting as early as ≤ 12 months of age without significant cardiac involvement, or later during early childhood through late adulthood, sometimes as late as the 6th decade of life (Gungor & Reuser, 2013; Kishnani & Howell, 2004; Montagnese et al., 2015; van der Ploeg & Reuser, 2008). Across this continuum, patients can develop cardiac manifestations in the first year but not the cardiomyopathy noted in classic infantile Pompe disease (Slonim et al., 2000).

### 1.2 The *GAA* gene and Pompe disease

The *GAA* gene is responsible for the production of GAA protein. The occurrence of different pathogenic variants within the *GAA* gene causing various levels of GAA enzyme deficiency and abnormal enzyme activity (HP:0012379) influences disease severity and manifestations across the disease spectrum and is a primary contributing factor to the considerable variation seen in age of presentation, severity, and rate of progression (M. Kroos, Hoogeveen‐Westerveld, van der Ploeg, & Reuser, 2012; M. Kroos et al., 2008; M. A. Kroos et al., 2007; Montagnese et al., 2015; Montalvo et al., 2006). Complete deficiency of the GAA enzyme, resulting in enzyme activity < 1% of normal, is associated with classic infantile Pompe disease. Residual enzyme activity is more variable in other patient groups, and generally is not more than 30% of normal (Kishnani & Hwu, 2017; van der Ploeg & Reuser, 2008). To date, more than 400 pathogenic variants, in addition to numerous benign variants and variants of unknown significance (VUS), in the *GAA* gene have been reported (ClinVar, 2017; Erasmus MC University Medical Center, 2017; Exome Aggregation Consortium (ExAC), 2017; Genome Aggregation Database (gnomAD), 2017; Leiden Open Variation Database (LOVD), 2018; The Human Genome Mutation Database (HGMD), 2017). Confounding genetic and nongenetic factors not yet identified seem to contribute to the clinical diversity seen in phenotypes, including among family members with the same pathogenic *GAA* genotypes (De Filippi et al., 2014; M. A. Kroos et al., 2007; Slonim et al., 2007; Wens et al., 2013). A common aspect for these patients is the presence of at least one variant supporting the production of some residual GAA activity.

Identification of variants in the *GAA* gene provides valuable information for confirming diagnosis or carrier status; determining variant‐phenotype and genotype–phenotype correlations; and genetic counseling. In addition, when standard biochemical assays are not available, sequence variant identification may be used to predict cross‐reactive immunologic material (CRIM) status, which can influence treatment decisions (Bali et al., 2012; Kishnani et al., 2010).

### 1.3 The Pompe Registry

The Pompe Registry is a long‐term, multinational, and observational program (NCT002314000) designed to improve understanding of the natural history and outcomes of patients with Pompe disease. It started in 2004 and is sponsored and administered by Sanofi Genzyme (Cambridge, MA). Patients with a confirmed diagnosis of Pompe disease can be enrolled by physician investigators worldwide regardless of age, clinical manifestations, or treatment status. Participation by physicians and patients is completely voluntary. Patients provide appropriate written informed consent for their health information to be submitted to the Registry and for their data to be used in aggregate analyses. Treating physicians determine the frequency and type of assessments according to the individual patient's need for standard medical care and follow‐up. This detailed patient information is collected in the Pompe Registry database. Because of the voluntary and observational nature of the program, the frequency and types of assessments reported for each patient will vary. Physicians can report their patients’ characteristics, including gender; age; clinical characteristics; and diagnostic parameters, including methods of diagnosis and the results of enzymatic and genetic testing. The Pompe Registry can be accessed only by participating physicians. In accordance with the consent provided by patients, information gathered in the Pompe Registry can only be shared to the general public through peer‐reviewed publications. This is also the mechanism by which the Registry can provide information for other genetic and phenotype databases. The Pompe Registry is the largest global repository of data for Pompe disease and a valuable resource for the medical community. Information on the Registry can be found at https://www.registrynxt.com/ and also at https://clinicaltrials.gov.

We have analyzed reported aggregate patient information in the Pompe Registry to first determine the number, frequency, and characteristics of all *GAA* sequence variants that were reported into the Registry database as candidate disease‐associated variants among patients diagnosed with Pompe disease on the basis of clinical features and GAA deficiency. The present analysis is a foundational report and is the first of a series of planned analyses on variant data from the Pompe Registry. It focuses on the geographic distribution and frequencies of the variants, including the novel, as yet unpublished, variants. While Pompe disease is a clinical spectrum, analysis of the variant‐phenotype and preliminary genotype–phenotype correlations is based on the assignment of variants to three groups of patients characterized by age of symptom onset with or without cardiomyopathy (HP:0001638).

## 2 METHODS

### 2.1 Patient eligibility

Patients with the following data in the Pompe Registry were eligible for inclusion: Pompe disease diagnosis reported as confirmed by enrolling physicians and with ≥ 1 documented pathogenic variant. Patients were excluded if dates of diagnosis and/or symptom onset were missing.

### 2.2 Data collection of patient characteristics and variant information

Demographic and clinical characteristics were collected on the Registry's case report form (CRF). Whether patients were identified through newborn screening was not part of this analysis. The CRF does not provide information regarding methods for CRIM‐status determination. Ethnicity and race are self‐reported on the CRF since the Registry does not perform ancestry analyses to confirm what was reported. General sibling information collected in the Registry is reported. However, consanguinity is not collected and an analysis of linked sibling data was not performed. *GAA* variant and phenotype information from patient records in the Pompe Registry was evaluated. Healthcare teams entering patient information at Registry sites are instructed to enter the preferred c.DNA nomenclature in a free text field in the Registry's CRF. Protein information is deduced from c.DNA, but RNA information for variants is not collected by the Registry.

### 2.3 Informed consent and patient privacy

Each independent site is responsible for obtaining patients’ informed written consent to submit their health information to the Registry, and to use and disclose this information in aggregate analyses. The Registry protocol, informed consent form, and any locally required authorization documents to send patient information to the Registry are reviewed and approved by the local fully constituted Institutional Review Board (IRB) or Independent Ethics Committee (IEC) unless the site provides the Registry with documentation that approval is not required or has been waived by a particular IRB/IEC.

Informed consent is required to share patient data submitted to the Pompe Registry. Therefore, only novel *GAA* variants for which appropriate updated patient consent was available have been submitted to the Single Nucleotide Polymorphism Database (dbSNP; submission ID: SUB4205047; http://www.ncbi.nlm.nih.gov/SNP/) and the Leiden Open Variation Database (LOVD; https://www.lovd.nl/).

### 2.4 Variant nomenclature and standardization

Variant data are reported by individual sites, and errors and discrepancies with how individual *GAA* sequence variants were reported are possible. Variants also may be entered in different ways as a result of variable interpunction, letter spacing, or software‐directed automatic use of upper or lower case. Extensive reviews and consultations with Registry sites were conducted and concerted efforts made to standardize the nomenclature for the reported variants to allow meaningful analyses and interpretation of results.

The Human Genome Variation Society (HGVS) recommendations were used to standardize nomenclature for variants reported in the Registry (den Dunnen et al., 2016). For example, duplicated nucleotides after the “dup” were excluded (e.g., "c.258dupC" was standardized to "c.258dup"), as were deleted nucleotides after the "del" (e.g., "c.1354_1372del19" was standardized to "c.1354_1372del"). In addition, specified nucleotides were included for all insertions and deletions/insertions (e.g., c.2741delins was standardized to c.2741delinsGAC). Location in the *GAA* gene, variant type, protein identifications for variants, and pathogenicity for novel variants was predicted based on the c.DNA data available in the Registry (see Acknowledgments and Supporting Information). Identified variants that do not cause disease but that cause low‐GAA activity when measured in enzyme assays, defined as pseudodeficiency variants (Labrousse et al., 2010; Tajima et al., 2007) are described. The following reference sequences were used to specify *GAA* variants: NM_000152.5 for the coding region conform the Ensembl sequence of the forward strand on Chromosome 17: 80,101,556‐80,119,879 [GRCh38:CM000679.2]; protein id [NP_000143.2], and intronic variants according to the same gene locus Ensembl sequence [ENSG00000171298] [LRG_673][NCBI Reference Sequence: NG_009822.1][NC_000017.10]. Relevant information can be readily accessed via the HGNC Symbol report for GAA (HGNC 2018, 2018).

### 2.5 Identification of novel variants

Variants were classified as “novel” if they were reported in the Registry but not in the following publicly available sources: Current listings in *GAA* variant databases (Duke University Medical Center, 2017; Erasmus MC University Medical Center, 2017) or other sequence and variant databases (Exome Aggregation Consortium (ExAC), 2017; Genome Aggregation Database (gnomAD), 2017; Genome Project Consortium, 1000, 2017; Leiden Open Variation Database (LOVD), 2018; NHLBI GO Exome Sequencing Project (ESP), 2017), public archives (ClinVar, 2017), database resources of the National Center for Biotechnology Information (NCBI Resource Coordinators, 2016), or through searches of other sources and databases (including unpublished data; and data on file, Sanofi Genzyme) through June 2017. Unique is defined here as meaning different or distinct individual variants that were reported in the Registry analysis data set. For example, if 25 reported variants are c.525del and 7 are c.307T>G, then the number of reported variants is 32 and the number of *unique* variants reported is 2. All *GAA* variants reported in this study (other than novel variants) are either linked with Pompe disease http://www.pompecenter.nl (>molecular aspects>Pompe variant database) or tentatively marked as candidate disease‐associated variants as they were found in the DNA of patients who were diagnosed with Pompe disease. Because the Registry CRF does not inquire about the cis/trans occurrence of *GAA* variants and also does not include confirmation of trans‐position by parent investigation, the likely impact of novel variants was predicted using bioinformatics algorithms, such as PROVEAN (Choi, Sims, Murphy, Miller, & Chan, 2012), SIFT (Sim et al., 2012), PolyPhen (Adzhubei et al., 2010), and FATHMM (Shihab et al., 2015). The programs were deemed appropriate and chosen based on a number of criteria in the ACMG Standards and Guidelines for Interpretation of Sequence Variants (Richards et al., 2015) and Guidelines for Prediction Tools for Genetic Variation Analysis (Vihinen, den Dunnen, Dalgleish, & Cotton, 2012). These methods are publicly available for research and were chosen based on their proven performances. Also, as recommended, because each sequence interpretation program has inherent strengths and weaknesses and sensitivities depending on the algorithm used, multiple predictive methods were used to decrease overpredictions and increase more accurate conclusions and predictions of impact (Vihinen et al., 2012; see Supporting Information online). Criteria for classifying variants based on their severity scores and evidence of pathogenicity (viz., benign, likely benign, variant of uncertain significance [VUS], likely pathogenic, and pathogenic) as outlined in the ACMG guidelines for the interpretation of sequence variants were used for the classification of novel variants by predicted pathogenicity (Richards et al., 2015). Variants could be classified as VUS due to either insufficient evidence or conflicting evidence and are identified as such. The effects of all previously published variants, as currently listed in *GAA* variation databases, were not re‐analyzed.

### 2.6 Patient phenotypic subgroup classification

Patients were classified into one of three groups based on the reported age at first sign or symptom onset and reported the presence or the absence of cardiomyopathy: Group A, onset of symptoms ≤ 12 months of age *with* cardiomyopathy (patients typically classified as classic infantile Pompe disease); Group B, onset of symptoms ≤ 12 years of age (includes patients with onset of symptoms ≤ 12 months of age *without* cardiomyopathy in the first year of life and not included in Group A); and Group C, onset of symptoms > 12 years of age. The presence of cardiomyopathy was obtained from patient records as reported on the Registry CRF and may not reflect the exact onset. The patient classifications were based on the criteria described in previous Registry publications (Kishnani et al., 2013, 2014) to allow for meaningful analysis, interpretation, and explanation of results of the reported Registry data. Clinical features of phenotypes were described using available Human Phenotype Ontology (HPO) terms and identification numbers (Kohler et al., 2019; The Human Phenotype Ontology (HPO), 2019).

To provide further clinically relevant information, homozygous genotypes were used to further rate a subset of variants by degree of severity.

### 2.7 Statistical analyses

Descriptive statistics were used to summarize demographic and variant information as this was a foundational report intended to provide an initial overview of variants reported among patients in the Pompe Registry. Thus, since there were no a priori hypotheses, no formal inferential statistical tests were conducted. Frequencies reported in 1 to < 5 patients are reported as < 5 to protect patient privacy. All analyses were conducted using SAS 9.4 (SAS Institute Inc., Cary, NC).

## 3 RESULTS

### 3.1 Demographics

As of July 2017, 1,753 consented patients were enrolled in the Pompe Registry. Based on inclusion criteria, data were analyzed for 1,079 patients from 26 countries in five geographic regions (Europe, North America, Asia‐Pacific, Latin America, and the Middle East).

Patient demographics and baseline characteristics are shown in Table 1. Of note, patients from Latin America had to provide updated consent for continued participation in the Registry. Therefore, only the subset of enrolled Registry patients from Latin America with updated consent when data were collected (July 2017) are included. Of the 1,079 patients with evaluable sequence variant information, 190 were classified as Group A, 238 as Group B, and 651 as Group C. More than half (58.0%) of all patients were from Europe. The majority (71.2%) were self‐reported as Caucasian. Overall, and for each group, approximately half were female. Of the 257 patients reported to have siblings diagnosed with Pompe disease, 26 were in Group A, 43 in Group B, and 188 in Group C.

**Table 1:** Patient demographics and baseline clinical characteristics

	All patients	Group A^a^	Group B^b^	Group C^c^
Patients, *N*	1,079	190	238	651
Geographic region				
Europe	626 (58.0%)	67 (35.3%)	128 (53.8%)	431 (66.2%)
North America	343 (31.8%)	87 (45.8%)	77 (32.4%)	179 (27.5%)
Asia‐Pacific	93 (8.6%)	29 (15.3%)	29 (12.2%)	35 (5.4%)
Latin America	9 (0.8%)	<5	<5	6 (0.9%)
Middle East	8 (0.7%)	5 (2.6%)	<5	0
Number of countries	26	18	18	22
Race				
Caucasian	768 (71.2%)	88 (46.3%)	165 (69.3%)	515(79.1%)
Black	42 (3.9%)	26 (13.7%)	5 (2.1%)	11 (1.7%)
Asian	97 (9.0%)	31 (16.3%)	37 (15.5%)	29 (4.5%)
Other^d^	18 (1.7%)	7 (3.7%)	6 (2.5%)	5 (0.8%)
Unknown	154 (14.3%)	38 (20.0%)	25 (10.5%)	91 (14.0%)
Gender				
Males	527 (48.8%)	92 (48.4%)	130 (54.6%)	305 (46.9%)
Females	552 (51.2%)	98 (51.6%)	108 (45.4%)	346 (53.1%)
Age at symptom onset (years)				
Mean (SD)	23.0 (20.1)	0.2 (0.2)	4.0 (4.0)	36.5 (14.0)
Median (min, max)	20.4 (0.0,75.8)	0.2 (0.0,0.9)	2.2 (0.0,12.0)	36.9 (12.1,75.8)
Age at diagnosis (years)				
Mean (SD)	28.9 (22.6)	0.3 (0.2)	13.9 (16.7)	42.8 (15.1)
Median (min, max)	32.1 (0.0,82.5)	0.3 (0.0,1.3)	4.6 (0.0,69.2)	43.0 (0.4,82.5)
CRIM status				
CRIM positive, *N* (%)	175 (16.2%)	95 (50.0%)	38 (16.0%)	42 (6.5%)
CRIM negative, *N* (%)	42 (3.9%)	40 (21.1%)	<5	0
CRIM unknown *N* (%)	862 (79.9%)	55 (28.9%)	198 (83.2%)	609 (93.5%)
Siblings diagnosed with Pompe disease (*N*)	257	26	43	188
Number diagnosed				
1	186	22	31	133
2	44	<5	8	35
≥3	12	0	<5	11
Missing	15	<5	<5	9

Abbreviation: CRIM, cross‐reactive immunologic material.

Denominators of percentages are based on *N* values for each group (all patients: *N* = 1,079; Group A: *N* = 190; Group B: *N* = 238; Group C: *N* = 651) unless otherwise specified. Frequencies reported in the Pompe Registry in fewer than five patients are reported as < 5 to protect patient privacy.

^a^ Group A: Onset of symptoms ≤ 12 months of age *with* cardiomyopathy (patients classified as classic infantile Pompe disease). Group A also may include a subset of patients with less severe cardiomyopathy and slower disease progression.

^b^ Group B: Onset of symptoms ≤ 12 years of age (includes patients with onset of symptoms ≤ 12 months of age *without* cardiomyopathy and not included in Group A).

^c^ Group C: Onset of symptoms > 12 years of age.

^d^ Native Hawaiian or other Pacific Islander or multiple race categories.

For Group A, mean age at symptom onset was 0.2 years (2.4 months) and mean age at diagnosis was 0.3 years (3.5 months). In contrast, for Group B, mean age at symptom onset was 4.0 years (median age: 2.2 years) and mean age at diagnosis was 13.9 years (median age: 4.6 years), with a diagnostic gap between symptom onset and a confirmed diagnosis of greater than 10 years for some patients. Group C had a mean age at symptom onset of 36.5 years and mean age at diagnosis of 42.8 years (Table 1).

Table 1 also lists the CRIM status of patients, whereby CRIM indicates any form of GAA protein, catalytically active or inactive, that is detected by immunologic procedures. Among the 135 Group A patients with CRIM status reported, 95 (70.4%) were CRIM‐positive and 40 (29.6%) were CRIM‐negative. CRIM status was unknown for 28.9% of the Group A.

### 3.2 Variants and their geographic distribution by group

A total of 2,075 (1,205 exonic/ 870 intronic) *GAA* variants were reported among the 1,079 patients (Table 2). Of these, 392 variants were unique (as defined in Methods), 80 being novel (Table 3), and 312 variants (Table S1) being known. Two or more variants were reported in the large majority (*n* = 969) of patients (Table 2). Nineteen patients (7 in Group A; 5 in Group B; and 7 in Group C) had three variants reported, and four patients had four (2 in Group A; and 1 in Groups B and C each). Among patients with two or more variants were 17 with the common pseudodeficiency variant, c.1726G>A frequently found in Asian populations (M. A. Kroos et al., 2008; Kumamoto et al., 2009; Yang et al., 2011) and < 5 with the pseudodeficiency allele c.271G>A, frequent in Caucasians (Swallow et al., 1989; van Diggelen et al., 2009; data not shown).

**Table 2:** Frequency of variants among patients with variant information by phenotypic subgroups

Parameter	All patients	Group A^a^	Group B^b^	Group C^c^
Number of variants, *N* ^d^ ^,^ ^e^	2,075	373	459	1,243
Novel variants, *N* (%)^f^	94 (4.5%)	18 (4.8%)	17 (3.7%)	59 (4.7%)
Unique novel variants,^e^ ^,^ ^f^ *N* (%)	80 (3.9%)	17 (4.6%)	16 (3.5%)	49 (3.9%)
Known variants, *N* (%)^e^	1,981 (95.5%)	355 (95.2%)	442 (96.3%)	1,184 (95.3%)
Unique known variants, *N* (%)	312 (15.0%)	135 (36.2%)	146 (31.8%)	196 (15.8%)
Patients with any variant entry, *N*	1,079	190	238	651
Patients with ≥2 variant entries, *N* ^d^	969	172	214	583
Patients with 1 variant entry, *N*	110	18	24	68

^a^ Group A: Onset of symptoms ≤ 12 months of age *with* cardiomyopathy (patients typically classified as having classic infantile Pompe disease). Group A also may include a subset of patients with less severe cardiomyopathy and slower disease progression.

^b^ Group B: Onset of symptoms ≤ 12 years of age (includes patients with onset of symptoms ≤ 12 months of age *without* cardiomyopathy and not included in Group A).

^c^ Group C: Onset of symptoms > 12 years of age.

^d^ Some patients have a combination of more than 2 variants.

^e^ Known variants are defined as having been previously published or identified as described in the Methods section. Unique is defined here as meaning different or distinct individual variants. The *N* is based on the total number of variants. Denominators for percentages are based on the total numbers of variants per patient groups (e.g., in Group A, 355 of known variants represent 95.2% of the total variants in this group).

^f^ Variants are classified as “novel” if they are reported in the Pompe Registry but not previously published or identified in publicly available sources (as described in the Methods) as of June 2017.

**Table 3:** Unique novel variants reported among patients by phenotypic subgroups

Location	DNA	Protein^a^	Phenotypic subgroup^b^	Prediction^c^			Pathogenicity^d^
				SIFT	Polyphen‐2 HVAR	FATHMM MKL	
exon2	c.295_314del	p.(Thr99ProfsTer40)	C				Pathogenic
exon2	c.541_545del	p.(Phe181AspfsTer6)	A				Pathogenic
exon3	c.665T>G	p.(Val222Gly)	A	D	PD	D	VUS – insufficient evidence
exon3	c.686G>C	p.(Arg229Pro)	C	T	B	T	VUS – insufficient evidence
exon3	c.692T>C	p.(Leu231Pro)	C	D	PD	D	VUS – insufficient evidence
intron3	c.692+1G>T	p.?	C	NA	NA	D	Pathogenic
intron3	c.693‐2A>C	p.?	A	NA	NA	D	Pathogenic
exon4	c.759del	p.(Ser254ArgfsTer14)	C				Pathogenic
exon4	c.766_784del	p.(Tyr256SerfsTer6)	B				Pathogenic
exon5	c.878G>T	p.(Gly293Val)	C	D	PD	D	Likely pathogenic
exon5	c.930_932del	p.(Phe311del)	A				VUS – Conflicting evidence
exon5	c.950C>T	p.(Ala317Val)	B	D	PD	D	VUS – conflicting evidence
exon6	c.982_988del	p.(Leu328GlyfsTer62)	A				Pathogenic
exon6	c.994_995insTT	p.(Ser332PhefsTer61)	C				Pathogenic
exon6	c.1005_1006insGG	p.(Ile336GlyfsTer57)	C				Pathogenic
exon6	c.1057C>T	p.(Gln353Ter)	C	NA	NA	D	Pathogenic
exon7	c.1109G>A	p.(Gly370Asp)	C	D	PD	D	VUS – conflicting evidence
exon7	c.1114C>G	p.(His372Asp)	C	D	PD	D	Likely pathogenic
exon7	c.1114C>T	p.(His372Tyr)	C	D	PD	D	Likely pathogenic
exon7	c.1121G>A	p.(Cys374Tyr)	C	D	PD	D	Likely pathogenic
exon7	c.1127_1130del	p.(Trp376SerfsTer15)	B				Pathogenic
exon7	c.1129G>A	p.(Gly377Ser)	B	D	PD	D	Likely pathogenic
exon8	c.1201C>A	p.(Gln401Lys)	C	D	PD	D	VUS – insufficient evidence
exon8	c.1211A>C	p.(Asp404Ala)	B	D	PD	D	Likely pathogenic
exon8	c.1211A>T	p.(Asp404Val)	A	D	PD	D	Likely pathogenic
exon8	c.1231del	p.(Arg411GlyfsTer29)	B				Pathogenic
exon8	c.1242C>A	p.(Phe414Leu)	C	D	PD	D	VUS – insufficient evidence
exon8	c.1311_1312ins26	NA	A				Pathogenic
exon9	c.1378G>T	p.(Glu460Ter)	C	NA	NA	D	Pathogenic
exon9	c.1388_1406del	p.(Arg463ProfsTer8)	C				Pathogenic
exon9	c.1396dup	p.(Val466GlyfsTer40)	C				Pathogenic
exon9	c.1409A>G	p.(Asn470Ser)	C	D	PD	D	VUS – insufficient evidence
exon10	c.1477C>T	p.(Pro493Ser)	B	D	PD	D	VUS – insufficient evidence
exon10	c.1507del	p.(Val503TrpfsTer17)	A				Pathogenic
exon10	c.1526A>T	p.(Gln509Leu)	C	T	PD	D	VUS – insufficient evidence
intron10	c.1551+3A>T	p.?	C				VUS – insufficient evidence
exon11	c.1559A>G	p.(Asn520Ser)	C	D	PD	D	VUS – insufficient evidence
exon12	c.1670T>G	p.(Ile557Ser)	C	D	PD	D	Likely pathogenic
exon12	c.1681_1699dup	p.(Thr567LysfsTer75)	C				Pathogenic
exon12	c.1688A>T	p.(Gln563Leu)	C	D	PD	D	Likely pathogenic
exon12	c.1721T>C	p.(Leu574Pro)	C	D	PD	D	VUS – insufficient evidence
intron12	c.1754+1dup	p.?	C				Pathogenic
exon13	c.1822del	p.(Arg608AspfsTer88)	C				Pathogenic
exon13	c.1825T>G	p.(Tyr609Asp)	C	D	PD	D	Likely Pathogenic
exon13	c.1839 G>C	p.(Trp613Cys)	B	D	PD	D	VUS – insufficient evidence
exon13	c.1847dup	p.(Asp61GlufsTer20)	C				Pathogenic
exon13	c.1876_1878del	p.(Ser627del)	C				VUS – insufficient evidence
exon14	c.1895T>C	p.(Leu632Pro)	C	D	PD	D	VUS – insufficient evidence
exon14	c.1944_1950del	p.(Phe649AlafsTer45)	B,C				Pathogenic
exon14	c.1961C>G	p.(Ser654Ter)	C	NA	NA	T	Pathogenic
exon14	c.2004C>A	p.(Tyr668Ter)	A	NA	NA	D	Pathogenic
exon14	c.2020C>T	p.(His674Tyr)	C	D	PD	D	VUS – insufficient evidence
intron14	c.2041‐2A>G	p.?	A				Pathogenic
exon15	c.2056_2057 delinsCC	p.(Ser686Pro)	A	T	B	T	VUS – insufficient evidence
exon15	c.2084dup	p.(Met695IlefsTer70)	C				Pathogenic
exon15	c.2096T>C	p.(Leu699Pro)	A	D	PD	D	VUS – insufficient evidence
exon15	c.2109del	p.(Tyr703Ter)	C				Pathogenic
exon15	c.2146G>C	p.(Ala716Pro)	B	D	PD	D	VUS – conflicting evidence
exon15	c.2153_2156delinsACGCCG	p.(Val718AspfsTer47)	B	T	B	T	Pathogenic
exon15	c.2182_2183del	p.(Phe728ProfsTer8)	C				Pathogenic
exon16	c.2205_2206insT	p.(Ser736Ter)	C				Pathogenic
exon16	c.2237G>T	p.(Trp746Leu)	B	D	PD	D	Likely pathogenic
exon16	c.2240G>A	p.(Gly747Glu)	C	D	PD	D	VUS – insufficient evidence
exon16	c.2258_2259insC	p.(Val755SerfsTer41)	C				Pathogenic
exon16	c.2261dup	p.(Val755LysfsTer10)	C				Pathogenic
intron16	c.2331+101del	p.?	C				VUS – conflicting evidence
exon17	c.2407C>T	p.(Gln803Ter)	C	NA	NA	D	Pathogenic
exon17	c.2459_2461del	p.(Ala820del)	A,C				VUS – conflicting evidence
exon17	c.2460dup	p.(Gly821TrpfsTer63)	B				Pathogenic
exon18	c.2480A>G	p.(Gln827Arg)	B	D	PD	D	VUS – insufficient evidence
exon18	c.2515C>T	p.(Gln839Ter)	A	NA	NA	D	Pathogenic
exon18	c.2584G>A	p.(Gly862Arg)	B	D	PD	D	VUS – insufficient evidence
exon18	c.2619C>G	p.(Tyr873Ter)	C	NA	NA	D	Pathogenic
exon19	c.2655_2656del	p.(Val886GlufsTer2)	C				Pathogenic
exon19	c.2720T>C	p.(Leu907Pro)	B	D	PD	D	VUS – insufficient evidence
exon19	c.2740dup	p.(Gln914ProfsTer104)	A				Pathogenic
exon19	c.2742dup	p.(Gln915AlafsTer103)	A				Pathogenic
exon19	c.2757del	p.(Asn919LysfsTer24)	C				Pathogenic
intron19	c.2800‐1G>C	p.?	A	NA	NA	D	Pathogenic
exon20	c.2845_2847del	p.(Val949del)	C				VUS – insufficient evidence

Abbreviations: B, benign; D, damaging; PD, probably damaging; T, tolerated; NA, information not available.

^a^ The protein is provided where data are available. p.? is used to indicate that the impact on the protein is not known.

^b^ Group A: Onset of symptoms ≤ 12 months of age with cardiomyopathy (patients classified as classic infantile Pompe disease) [Group A also may include a subset of patients with less severe cardiomyopathy and slower disease progression]; Group B: Onset of symptoms ≤ 12 years of age (includes patients with onset of symptoms ≤ 12 months of age without cardiomyopathy and not included in Group A); Group C: Onset of symptoms > 12 years of age.

^c^ Variants without Sift, Polyphen‐2 HVAR, or FATHMM‐MKL signature were not investigated as the nature of the variant predicted a damaging effect in all cases.

^d^ Pathogenicity is based on criteria for severity provided in the ACMG guidelines for interpretation of sequence variants (Richards et al., 2015).

Only one variant was reported in the Registry for 110 patients: 18 in Group A, 24 in Group B, and 68 in Group C (Table 2). Among these 110 patients, diagnosis was reported to have been made by a combination of DNA analysis and GAA enzyme assays, which include measurement of enzyme activity in blood‐based assays (lymphocytes and leukocytes), fibroblasts, and skeletal muscle tissue in most (82.7%) patients and by DNA analysis only in 17.3% of patients.

Table 4 provides an overview of the five most common variants across the clinical spectrum by patient group globally and regionally as reported in the Pompe Registry. Globally, eight different variants represent most of the reported variants. Two of these eight variants (c.525del and c.2481+102_2646+31del) are not group‐specific, and Groups B and C share the same common variants. Among Group A, the variant c.2560C>T is the most common globally and in both North America and Latin America and is among the five most common in Europe but not in Asia‐Pacific and the Middle East. The variant c.1935C>A was the second most common globally and the most common in Asia‐Pacific but was not frequently reported in any other region. Also, in Groups B and C, c.‐32‐13T>G is the most commonly reported variant globally as well as in Europe and North America, and in Group C only in Latin America, but not in Asia‐Pacific and the Middle East.

**Table 4:** The five most commonly reported variants among patients by phenotypic subgroup across the clinical spectrum of Pompe disease globally and by geographic region

Group A^a^		Group B^b^		Group C^c^	
DNA	Protein	DNA	Protein	DNA	Protein
**Global**					
***N* = 373 variants**		***N* = 459 variants**		***N* = 1,243 variants**	
c.2560C>T (*n* = 28)	p.(Arg854Ter)	c.‐32‐13T>G (*n* = 128)	p.?	c.‐32‐13T>G (*n* = 561)	p.?
c.1935C>A (*n* = 27)	p.(Asp645Glu)	c.2481+102_2646+31del (*n* = 20)	p.(Gly828_ Asn882del)	c.525del (*n* = 71)	p.(Glu176ArgfsTer45)
c.525del (*n* = 21)	p.(Glu176ArgfsTer45)	c.525del (*n* = 19)	p.(Glu176ArgfsTer45)	c.2481+102_2646+31del (*n* = 46)	p.(Gly828_ Asn882del)
c.2481+102_2646+31del (*n* = 14)	p.(Gly828_Asn882del)	c.2238G>C (*n* = 18)	p.(Trp746Cys)	c.2238G>C (*n* = 20)	p.(Trp746Cys)
*c.1726*G>A *(n = 11)* ^d^	p.(Gly576Ser)	c.307T>G (*n* = 9)	p.(Cys103Gly)	c.307T>G (*n* = 18)	p.(Cys103Gly)
**Europe**					
***N* = 125 variants**		***N* = 245 variants**		***N* = 822 variants**	
c.525del (*n* = 12)	p.(Glu176ArgfsTer45)	c.‐32‐13T>G (*n* = 88)	p.?	c.‐32‐13T>G (*n* = 398)	p.?
c.2481+102_2646+31del (*n* = 9)	p.(Gly828_Asn882del)	c.2481+102_2646+31del (*n* = 11)	p.(Gly828_ Asn882del)	c.525del (*n* = 55)	p.(Glu176 ArgfsTer45)
c.2237G>A (*n* = 7)	p.(Trp746Ter)	c.525del (*n* = 9)	p.(Glu176 ArgfsTer45)	c.2481+102_2646+31del (*n* = 25)	p.(Gly828_ Asn882del)
c.2560C>T (*n* = 7)	p.(Arg854Ter)	c.307T>G (*n* = 8)	p.(Cys103Gly)	c.307T>G (*n* = 14)	p.(Cys103Gly)
c.1933G>A (*n* = 5)	p.(Asp645Asn)	c.1933 G>A (*n* = 5)	p.(Asp645Asn)	c.1655T>C (*n* = 10)	p.(Leu552Pro)
**North America**					
***N* = 172 variants**		***N* = 148 variants**		***N* = 342 variants**	
c.2560C>T (*n* = 19)	p.(Arg854Ter)	c.‐32‐13T>G (*n* = 40)	p.?	c.‐32‐13T>G (*n* = 154)	p.?
c.525del (*n* = 9)	p.(Glu176ArgfsTer45)	c.525del (*n* = 10)	p.(Glu176 ArgfsTer45)	c.2481+102_2646+31del (*n* = 21)	p.(Gly828_ Asn882del)
c.2297A>C (*n* = 6)	p.(Tyr766Ser)	c.2481+102_2646+31del (*n* = 9)	p.(Gly828_ Asn882del)	c.525del (*n* = 16)	p.(Glu176 ArgfsTer45)
c.1799G>A (*n*<5)	p.(Arg600His)	c.2560C>T (*n* = 5)	p.(Arg854Ter)	c.1655T>C (*n* = 5)	p.(Leu552Pro)
c.1844G>A (*n*<5)	p.(Gly615Glu)	c.1082C>T (*n*<5)	p.(Pro361Leu)	c.2560C>T (*n* = 5)	p.(Arg854Ter)
**Asia‐Pacific**					
***N* = 62 variants**		***N* = 58 variants**		***N* = 69 variants**	
c.1935C>A (*n* = 26)	p.(Asp645Glu)	c.2238G>C (*n* = 15)	p.(Trp746Cys)	c.2238G>C (*n* = 19)	p.(Trp746Cys)
c.1726G>A (*n* = 11)^d^	p.(Gly576Ser)	c.2662G>T (*n* = 5)	p.(Glu888Ter)	c.1935C>A (*n* = 7)	p.(Asp645Glu)
c.1411_1414del (*n*<5)	p.(Glu471ProfsTer5)	c.1634C>T (*n*<5)	p.(Pro545Leu)	c.1726G>A (n<5)^d^	p.(Gly576Ser)
c.1843G>A (*n*<5)	p.(Gly615Arg)	c.1935C>A (*n*<5)	p.(Asp645Glu)	c.1822C>T (*n*<5)	p.(Arg608Ter)
c.1465G>A (*n*<5)	p.(Asp489Asn)	c.1726G>A (*n*<5)^d^	p.(Gly576Ser)	c.1843G>A (*n*<5)	p.(Gly615Arg)
**Latin America**					
***N*<5 variants**		***N*<5 variants**		***N* = 10 variants**	
c.2560C>T (*n*<5)	p.(Arg854Ter)	c.1556T>C (*n*<5)	p.(Met519Thr)	c.‐32‐13T>G (*n* = 7)	p.?
c.1912G>T (*n*<5)	p.(Gly638Trp)	c.1408_1410del (*n*<5)	p.(Asn470del)	c.1465G>A (*n*<5)	p.(Asp489Asn)
c.2481+102_2646+31del (*n*<5)	p.(Gly828_Asn882del)	—	—	c.1832G>A (*n*<5)	p.(Gly611Asp)
—	—	—	—	c.2560C>T (*n*<5)	p.(Arg854Ter)
**Middle East**					
***N* = 10 variants**		***N*<5 variants**		***N* = 0**	
c.340_341insT (*n* = 6)	p.(Lys114fsTer32)	c.1064T>C (*n*<5)	p.(Leu355Pro)	—	—
c.2015G>A (*n*<5)	p.(Arg672Gln)	c.1210G>A (*n*<5)	p.(Asp404Asn)	—	—
c.1799G>A (*n*<5)	p.(Arg600His)	c.2015G>A (*n*<5)	p.(Arg672Gln)	—	—
c.2056_2057delinsCC (*n*<5)	NA	c.2105G>A (*n*<5)	p.(Arg702His)	—	—

*Note*. Variant frequencies reported in the Pompe Registry in fewer than five patients are reported as < 5 to protect patient privacy.

^a^ Group A: Onset of symptoms ≤ 12 months of age *with* cardiomyopathy (patients classified as classic infantile Pompe disease). Group A also may include a subset of patients with less severe cardiomyopathy and slower disease progression.

^b^ Group B: Onset of symptoms ≤ 12 years of age (includes patients with onset of symptoms ≤ 12 months of age *without* cardiomyopathy and not included in Group A).

^c^ Group C: Onset of symptoms > 12 years of age.

^d^ c.1726G>A is a well‐known pseudodeficiency variant and is linked to other variants (see main text).

Other variants predicted to affect correct splicing or causing in‐frame smaller or larger structural changes (encompassing 1–54 amino acids) were equally frequent in all three groups (data not shown). The functional impact of most novel variants cannot be foreseen. This is true for missense variants, small in‐frame deletion or insertion variants, and variants in or near splice sites that have either no effect, a partial effect, or a detrimental effect on GAA synthesis and function. The ACMG guidelines for classifying the novel variants based on severity were followed (see section 3.3 novel variants for results). For severity rating one can cautiously, in parallel, also build on the phenotype of the patients in which the variants were found. Thus, we have listed all individual variants reported in Group A in a separate Table 5. Each variant reported in Table 5 likely contributes to the classic infantile Pompe disease phenotype and is potentially very severe.

**Table 5:** All variants reported among patients within Group A in the Pompe Registry

Location	DNA
intron1	c.‐32–17_‐32–10delinsTCCCTGCTGAGCCTCCTACAGGCCTCCCG
exon2	c.40_47del; c.236_246del; c.258dup; c.266G>A; c.307T>G; c.340_341insT; c.352C>T; c.378_379del; c.525_526del; c.525del; **c.541_545del**
intron2	c.546+2_5del
exon3	c.572A>G; c.655G>A; **c.665T>G;** c.670C>T
intron3	**c.693‐2A>C**
exon4	c.716del; c.722_723del; c.784G>A; c.794del; c.854C>G
exon5	c.871C>T; c.872T>C; c.877G>A; c.925G>A; **c.930_932del;** c.947A>G; c.953T>C
exon6	**c.982_988del;** c.1000G>T; c.1062C>G; c.1064T>C
intron6	c.1075+13C>T
exon7	c.1082C>T; c.1099T>C; c.1115A>T; c.1129G>C; c.1157dup; c.1190C>T
intron7	c.1195–2A>G
exon8	c.1197_1208del; c.1210G>A; c.1211A>G; **c.1211A>T;** c.1221C>A; c.1281G>T; c.1286A>G; **c.1311_1312ins26**
exon8–15	c.1195‐18_2190‐20del
intron8	c.1327‐2A>G
exon9	c.1396G>T; c.1396del; c.1402A>T; c.1408_1410del; c.1411_1414del
intron9	c.1437+1G>A; c.1437+2T>C; c.1438–1G>C
exon10	c.1441T>C; c.1441del; c.1447G>A; c.1465G>A; c.1466A>G; c.1496G>A; **c.1507del;** c.1548G>A
intron10	c.1551+1G>T
exon11	c.1561G>A; c.1564C>G; c.1564C>T
intron11	c.1637–2A>G
exon12	c.1642G>T; c.1650dup; c.1654del; c.1655T>C; c.1703A>T; c.1705dup; c.1724A>C; c.1726G>A^a^; c.1735G>A
intron12	c.1754+1G>A; c.1754+2T>A
exon13	c.1796C>A; c.1798C>T; c.1799G>A; c.1802C>G; c.1802C>T; c.1822C>T; c.1841C>A c.1843G>A; c.1844G>A; c.1846G>A; c.1880C>T
exon14	c.1912G>T; c.1913G>A; c.1927G>A; c.1933G>A; c.1933G>C; c.1933G>T; c.1935C>A; c.1941C>G; c.1942G>A; c.1962_1964del; c.1979G>A**; c.2004C>A**; c.2015G>A; c.2023_2025del; c.2024_2026del; c.2040G>A
intron14	**c.2041‐2A>G**
exon15	c.2051C>T; **c.2056_2057delinsCC;** c.2078dup; **c.2096T>C;** c.2104C>T; c.2105G>T; c.2173C>T
exon16	c.2219_2220del; c.2227C>T; c.2236T>C; c.2237G>A; c.2238G>A; c.2274dup; c.2294G>A; c.2296T>A; c.2297A>C; c.2303C>T
intron16	c.2331+1G>A; c.2331+2T>A
exon17	c.2408_2426del; **c.2459_2461del**
intron17	c.2481+102_2646+31del
exon 18	c.2495_2496del; c.2501_2502del; c.2512C>T; **c.2515C>T;** c.2528T>C; c.2560C>T; c.2608C>T
intron18	c.2646+2T>A
exon19	c.2662G>T; c.2707_2709del; **c.2740dup;** c.2741delinsGAC; **c.2742dup;** c.2770T>C
intron19	**c.2800‐1G>C**
exon20	c.2815_2816del; c.2841_2842insT; c.2846T>A

*Note*. Variants in boldface indicate novel variants. They were reported in the Registry but not reported in the publicly available sources (see Methods) identified as of June 2017.

^a^ c.1726G>A is a well‐known pseudodeficiency variant and is linked to other variants (see main text).

### 3.3 Novel variants

**Figure 1:**
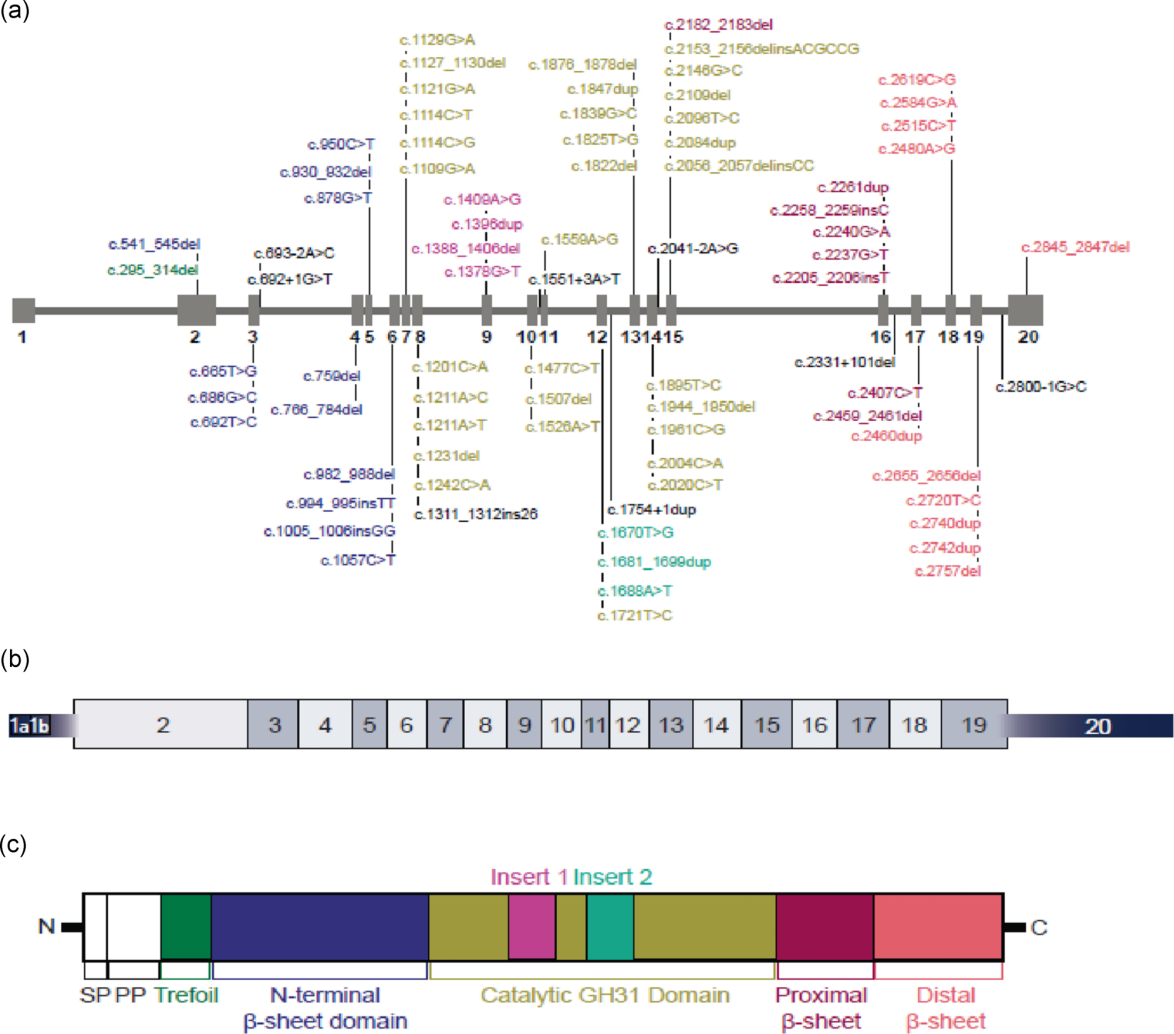
Location of novel variants in the *GAA* gene. (a) *GAA* gene with novel variants reported in the Pompe Registry. Variant listings are color coded to identify corresponding domain in the GAA protein (panel c). Variants listed in black text are either intronic or have no apparent protein‐level change. (b) GAA mRNA. (c) GAA protein

There were 94 (4.5%) novel variants reported (18 in Group A, 17 in Group B, and 59 in Group C). Of these, 80 are unique (Tables 2, 3). Most novel variants were reported in patients in Europe (66.7%) and North America (20.0%). As seen in Figure 1, these unique novel variants were located throughout the *GAA* gene (73 in exons; 7 in introns). All variant types were represented. Overall, substitution (missense) was the most frequent type of variant reported among the three groups, accounting for 40.0% (*n* = 32) of the 80 unique novel variants, followed by frameshift variants (33.8%; *n* = 27). Without knowing the precise impact of these variants on *GAA* function, there is good reason to assume that the majority of the novel variants have a pathogenic effect since they were identified in patients diagnosed with Pompe disease in absence of other known sets of disease‐causing *GAA* variants.

In addition, the impact of the novel variants was preliminarily predicted using the validated programs (Richards et al., 2015; Vihinen et al., 2012), SIFT, PolyPhen, or FATHMM for 47 of the 80 unique novel variants for which impact was not evident from their nature (Table 3; Supporting Information). The predicted pathogenicity based on the ACMG criteria for severity and impact (where available) for the 80 unique novel variants are reported in Table 3. Overall, 51.3% (*n* = 41) of the 80 variants were classified as pathogenic, 13.8% (*n* = 11) as likely pathogenic, and 35% (*n* = 28) as VUS. Of the 17 unique novel variants reported for Group A, 11 were classified as pathogenic, one as likely pathogenic, and five as VUS (Table 3). Among these 17 unique novel variants reported for Group A, frameshift variants were the most common type (35.3%; *n* = 6). Of these variants, two (c.541_545del and c.982_908del), present in exons 2 and 6, respectively, mapped to the N Terminal Beta Sheet domain; one (c.1507del) mapped to the catalytic GH31 domain; and two (c.2740dup and c.2742dup) mapped to the Distal Beta Sheet domain. Only one (c.1311_1312ins26), present at exon 8, mapped to the catalytic GH31 domain just before the start of insert 1 in the translated protein. No predictions of impact were available for these frameshift variants using bioinformatics algorithms. However, all were classified as pathogenic based on the ACMG's severity scores. Other variant types were reported in < 5 patients in Group A. Deletion (small) variants mapped to the N terminal Beta Sheet domain (c.930_932del), the catalytic GH31 domain (c.2056_2057del), and the Proximal Beta Sheet (c.2459_2461del, also present in Group C). All three variants were classified as VUS based on the ACMG guidelines. Splicing variants mapped to the N Terminal GH31 domain (c.693‐2A>C), the catalytic GH31 domain (c.2041–2A>G), and the Distal Beta Sheet (c.2800–1G>C). Substitution (missense) variants mapping to the N terminal Beta Sheet domain were predicted to be damaging by SIFT and FATHMM and probably damaging by Polyphen (c.665T>G). Three of these four variants (c.693–2A>C, c.2041–2A>G, and c.2800–1G>C) were predicted to be pathogenic; the fourth, c.665T>G was classified as VUS per the ACMG severity scoring. Those mapping to the catalytic GH31 domain (c.1211A>T and c.2096T>C) were predicted to be damaging by SIFT and FATHMM and probably damaging by Polyphen. As noted in Table 3, c.1211A>T was classified as likely pathogenic, and c.2096T>C as VUS. Substitution (nonsense) variants mapping to the catalytic GH31 domain (c.2004C>A) and Distal Beta Sheet (c.2515C>T) were both not predicted by SIFT and Polyphen but were predicted as damaging by FATHMM. Per the ACMG criteria, both variants were classified as pathogenic.

Of the 16 unique novel variants reported for Group B, six were classified as pathogenic, three as likely pathogenic, and seven as VUS (Table 3). Two variant types comprised all 16 unique novel variants reported in Group B: substitution (missense) variants (62.5%; *n* = 10) and frameshift (37.5%; *n* = 6). Among the 49 unique novel variants for Group C, 25 were classified as pathogenic, seven as likely pathogenic, and 17 as VUS (Table 3). The most frequent types were substitution (missense) variants (38.8%; *n* = 19) and frameshift (32.7%; *n* = 16). Table S1 describes the distribution of previously published (nonnovel) variants by the patient phenotypic subgroup. Of the 80 unique novel variants, 53 had appropriate patient consent and were entered into dbSNP (submission ID SUB4205047; http://www.ncbi.nlm.nih.gov/SNP/) and the Leiden Open Variation Database (LOVD; https://www.lovd.nl/; Table S2).

### 3.4 Homozygous patients

Homozygous patients are readily informative for genotype–phenotype analysis. Among 172 patients in Group A with ≥ 2 identified variants, 56 (32.6%) patients were homozygous (Table 6). Of these patients, 53.6% were CRIM‐positive and 44.6% were CRIM‐negative. Among patients with ≥ 2 identified variants in Groups B (*n* = 214) and C (*n* = 583), 12 (5.6%) and 17 (2.9%), respectively, were homozygous (Table 6). Notably, three homozygous variants (c.1843G>A, c.1933G>A, and c.1935C>A) were reported in both Groups A and B, and one (c.2481+102_2646+31del) in both Groups A and C.

**Table 6:** Variants reported in homozygosity for patients by phenotypic subgroup across the clinical spectrum of Pompe disease

Group A by CRIM status (*N* = 56 patients)^a^		Group B (*N* = 12 patients)^b^	Group C (*N* = 17 patients)^c^
CRIM‐positive (*n* = 30)	CRIM‐negative (*n* = 25)		
c.307T>G	c.340_341insT	c.‐32‐13T>G	c.‐32‐13T>G (*n*=13)
c.655G>A	c.525_526del	c.670C>T	c.‐32‐3C>A
c.877G>A	c.525del	c.1064T>C	c.‐32‐3C>G
c.925G>A	c.1195‐18_2190‐20del	c.1437G>A	c.1076‐22T>G
c.1195‐2A>G	c.1496G>A	c.1447G>A	c.2481+102_2646+31del^d^
c.1210G>A	c.1637‐2A>G	c.1843G>A^e^	
c.1561G>A	c.2237G>A	c.1933G>A^e^	
c.1564C>G	c.2495_2496del	c.1935C>A	
c.1726G>A	c.2560C>T	c.2530_2541del	
c.1799G>A	c.2608C>T	c.2744A>C	
c.1843G>A	c.2662G>T		
c.1844G>A	c.2740dup		
c.1933G>A	c.2741delinsGAC		
c.1933G>C	c.2742dup		
c.1935C>A (*n*=7)			
c.1942G>A			
c.2015G>A			
c.2104C>T			
c.2297A>C			
c.2481+102_2646+31del			
**CRIM unknown**			
c.2078dup			

*Note:* Frequencies of each variant are < 5, except where noted. Some variants were reported in more than 1 patient, and therefore the total numbers of variants listed in the table are less than the number of patients in each group.

^a^ Group A: Onset of symptoms =?12 months of age with cardiomyopathy (patients classified as classic infantile Pompe disease). Group A also may include a subset of patients with less severe cardiomyopathy and slower disease progression.

^b^ Group B: Onset of symptoms =?12 years of age (includes patients with onset of symptoms =?12 months of age without cardiomyopathy and not included in Group A).

^c^ Group C: Onset of symptoms >?12 years of age.

^d^ The reporting of this variant in Group C, which is typically found in Group A, may reflect that the information was entered incorrectly in the Registry or may be the result of a technical error in the genetic analysis.

^e^ Group classification is based on age of symptom onset and reported the presence of cardiomyopathy (as described above). Any delays in cardiac assessment or diagnosis could lead to a misclassification. These patients appear to have been misclassified, most likely due to the date that cardiac assessments were requested and/or results recorded in the Registry. If this happened after the cutoff age for Group A (=?12 months of age), then the patient would be analyzed as Group B.

## 4 DISCUSSION

### 4.1 Summary

We analyzed data for 1,079 patients with *GAA* gene variants across five geographic regions and 26 countries, as entered in the Pompe Registry, a long‐term, ongoing, multinational, and observational program. A total of 2,075 variants spanning most of the *GAA* gene were reported, with the exceptions of exon 1 (noncoding), deep intronic regions, and promoter and regulatory gene regions. For such reasons, *GAA* sequence analysis results may not always inform the entirety of genetic contribution.

Of 2,075 variants reported in the Pompe Registry, 80 unique variants were novel, according to extensive review and standardization of nomenclature following the HGVS guidelines (den Dunnen et al., 2016) that allow meaningful sharing and interpretation of results (den Dunnen et al., 2016; Duke University Medical Center, 2017; Erasmus MC University Medical Center, 2017; Exome Aggregation Consortium (ExAC), 2017; Genome Aggregation Database (gnomAD), 2017; Genome Project Consortium, 1000, 2017; NHLBI GO Exome Sequencing Project (ESP), 2017; The Human Genome Mutation Database (HGMD), 2017). Reporting these novel variants is especially relevant for the prediction of phenotypes associated with these variants. While our manuscript was in preparation, 11 of these 80 novel variants appeared in a report on *GAA* variants discovered in French patients with Pompe disease (Semplicini et al., 2018).

With regard to the severity of *GAA* variants, those identified in Group A (Table 5) are “very severe” as they were found in patients with very early onset of first symptoms (The Human Phenotype Ontology (HPO), 2019) and cardiomyopathy (HP:0001638), that is, patients with classic infantile Pompe disease. Homozygosity for any of these variants is expected to lead to this phenotype, and this expectation is largely confirmed by the data on homozygous genotypes presented in Table 6.

The homozygous variants in Groups B and C are group‐specific, except for the most common c.‐13‐32T>G variant, which is known to be associated with a very broad clinical spectrum (M. Kroos et al., 2012; M. Kroos et al., 2008; M. A. Kroos et al., 2007; Montalvo et al., 2006; Semplicini et al., 2018). While we recognize and acknowledge apparent discrepancies in the reported data across the three phenotypic subgroups, these generally were not very many overall and were not sufficient to impact our conclusions.

This genotype–phenotype analysis has predictive value for a much larger group of patients. Not only homozygosity, but also compound heterozygosity in any combination of Group A variants (Table 5) will in principle be associated with the classic infantile phenotype. Variants that occur in Groups B and C in compound heterozygosity with a Group A variant will in principle be associated with less severe and less progressive phenotypes than those encountered in Group A. These suppositions have to be confirmed by future genotype–phenotype analyses.

Other than through analysis of genotype–phenotype correlations, the severity of variants can also be deduced from functional studies and in silico prediction. Functional studies were performed in the past (M. Kroos et al., 2012; M. Kroos et al., 2008; Montalvo et al., 2006). Because they could not be performed in the context of this study, in silico prediction programs were used to analyze the effect of all novel variants without obvious effect (variants causing frameshifts, those introducing premature stop codons, and those within position −2 to +2 of splice sites were taken as damaging). Using these programs, all but four novel variants were scored as damaging by at least one, but in the majority by two, prediction programs (see Table 3 and Supporting Information). As expected based on genotype‐phenotype correlation, the novel variants in Group A were scored as damaging by at least one of the prediction programs, including the two substitution (missense) variants c.665T>G that mapped to the N terminal Beta Sheet domain of the protein and c.1211A>T that mapped to the catalytic GH31 domain. As shown in Table 3, the c.665T>G variant was classified as VUS (due to insufficient evidence) and c.1211A>T as likely pathogenic. The only discrepancy found was c.2056_2057delinsCC, which resulted in a predicted substitution at the protein level (p.(Ser686Pro)) and mapped to the catalytic GH31 domain of the protein. The impact of this variant was predicted as tolerable with both SIFT and FATHMM and benign using PolyPhen. This is unexpected since this variant was identified in Group A, the most severe disease category, and the introduction of a proline for a serine is likely to affect the protein folding especially since the particular substitution is mapping to the catalytic domain of the protein. For this one particular variant, the prediction appears to have failed, that is, no correlation could be made between the predicted severity and a phenotype. In addition, this variant was classified as VUS (due to insufficient evidence) based on ACMG severity scores. Of the remaining variants, two substitution (missense) variants — c.686G>C mapping to the N terminal Beta Sheet domain and c.1526A>T mapping to the catalytic GH31 domain and predicted to be possibly not damaging by preliminary in silico analysis — were from patients in Group C. Both of these variants were classified as VUS (due to insufficient evidence). Also, the nonsense variant c.1961C>G and the frameshift variant c.2153_2156delinsACGCCG both mapped to the catalytic GH31 domain. They were identified in Group C and Group B, respectively. The predicted tolerability of the two missense variants might be relevant with regard to the phenotype of the patients in which they were found. Tolerability of the nonsense and frameshift variants seems very unlikely. In addition, both variants were classified as pathogenic when scored using the ACMG guidelines. It is possible, therefore, that the second allelic variant of these patients may account for the Group B and Group C phenotypes. The predictions provided here were the initial preliminary look at the novel variants. Genotype‐phenotype analysis, functional analysis, and more detailed in silico analysis in accordance with the existing guidelines (Richards et al., 2015; Vihinen et al., 2012) are required to properly report and interpret these prediction results and will be part of future publications. However, this approach showed consistency in the in silico preliminary analysis matching to phenotype subgroup classifications for all prediction but one. Based on current insight and using the ACMG guidelines for variant classification by severity scores, we were able to predict the pathogenicity of 52 of the 80 unique novel variants. Of these 52 variants, 79% (*n* = 41) were scored as pathogenic and 21% (*n* = 11) as likely pathogenic. The remaining 28 variants were scored as VUS (due to either insufficient or conflicting evidence; Table 3).

It may be difficult to estimate the severity of variants for a number of reasons, including if their natures does not allow for this, which is the case for most intronic variants; if functional studies are lacking, particularly for substitution (missense) variants; if in silico predictions are multi‐interpretable; if the GAA activity of the patient and the clinical signs are borderline normal/abnormal; and if a second damaging variant is not detected. One or more of these situations may apply to the 1,079 cases that were entered in the Registry and analyzed in this study. The 110 (10.1%) patients with just one variant reported deserve special mention. Among these patients, diagnosis was reported to have been made by a combination of DNA analysis and GAA enzyme assays in most patients (82.7%) and therefore the second variant may be missing because only standard sequencing techniques are commonly used in most countries. Further testing such as copy number variation (CNV) analysis using techniques sensitive enough to determine small duplications and deletions within exons and regulatory regions of the *GAA* gene, such as quantitative polymerase chain reaction (qPCR) or paralog ratio testing (PRT), are not done routinely. In addition, *GAA* mRNA assay that would allow detection of other types of variants such as deep intronic variants also is not performed by many laboratories. However, the importance of finding two variants to confirm Pompe disease diagnosis is recognized, and current initiatives to improve testing are underway, mainly through academic collaboration. Increased efforts to expand this and incorporate it into standard practice, for example, through the issuance and updating of guidelines, such as those from the European Pompe Consortium (EPOC; van der Ploeg et al., 2017), are needed. Nineteen of the 110 patients with just one variant were reported to have been diagnosed based on clinical signs and DNA analysis only and not by GAA enzyme assay. Although these patients were reported to have Pompe disease, their GAA level was not reported and we therefore cannot be certain how their diagnosis was confirmed. While all patients enrolled in the Pompe Registry must have a reported confirmed Pompe disease diagnosis (see Methods), we acknowledge the possibility of the presence of a second, unidentified or unrecorded pathogenic variant or of misdiagnosed carriers. Likewise, additional unidentified pathogenic or unrecorded variants may be present in cases where there were discordant results regarding the groupings of patients.

Misdiagnosis may also play a role in the differentiation between Group A and B patients. While age of onset of symptoms is the basis of patient classification in our analysis, it is not the sole discriminating factor for the patient phenotypic subgroups. Rather, the presence of cardiomyopathy in patients under the age of 1 year is the distinguishing feature. We cannot exclude that Group A may include a subset of patients with less severe cardiomyopathy and slower disease progression and Group B a subset of patients with a delayed diagnosis of cardiomyopathy as previously reported in the literature (Kishnani, Steiner et al., 2006; Slonim et al., 2000). For instance, homozygous variants c.1843G>A, c.1933G>A, and 1935C>A, typically found in classic infantile Pompe disease, were reported in both Groups A and B (Table 6). Upon careful review, the patients in Group B appear to have had cardiomyopathy reported in the Registry after 12 months of age.

With regard to the frequencies of individual variants globally, it must be noted that the figures are determined by the commonness of certain variants in ethnic groups and by the number of patients that were diagnosed in the specified geographic regions. Among all variants reported in the Registry, the leaky splice site variant c.‐32‐13T>G was by far the most common. This variant is found mostly in regions where Caucasian populations are more abundant. Most of the patients in the Pompe Registry were Caucasian (71.2%) and from Europe (58.0%). This variant typically is in 80–90% of adult Caucasian patients as well as some children with slower progressive forms of Pompe disease (Groups B and C) (Herbert, Cope, Li, & Kishnani, 2018; M. Kroos et al., 2012; M. A. Kroos et al., 2007; Musumeci et al., 2015; Reuser et al., 2018; van Capelle et al., 2016; Zampieri et al., 2011). Of note, the Registry does not collect information on ethnic origins, migrations, or other population movements that would allow for more in‐depth interpretation. Also, c.525del and c.2481+102_2646+31del are frequent among patients in all three groups in Europe (comprised mainly of Caucasian populations) and North American (comprised of populations of more varied ethnic origins), but are not among the most common in the other regions, with the exception for c.2481+102_2646+31del, which is among the variants reported for Group A in Latin America (De Filippi et al., 2014; M. Kroos et al., 2012; Reuser et al., 2018). Populations in Latin America represent a number of different ethnicities, with significant groups of Caucasian inheritance but also significant contributions from African, Native American, and Asian populations (Llerena et al., 2009). In this report, both the small number of patients from Latin America, as well as a lack of confirmed ethnic background information collected in the Pompe Registry, would not allow for a more in‐depth analysis.

In Asia‐Pacific, c.1935C>A was reported most often, but was not among the top five reported in other regions. Nevertheless, this variant was the fourth most common globally due to the input of patients from Asia‐Pacific where it accounted for nearly half of reported variants in Group A. Not unexpectedly, c1726G>A also was common in Group A in this region since c.1935C>A and c.1726G>A are linked in cis in patients in the Asia‐Pacific. The variant is a common pseudodeficiency variant that reduces the activity of GAA substantially if present but does not lead to Pompe disease. The variant does not occur alone but is always linked to c.2065G>A, another variant not leading to disease‐causing GAA deficiency. Interestingly, c.2065G>A should have been reported with minimally the same frequency as c.1935C>A (Kumamoto et al., 2009; Labrousse et al., 2010). However, the numbers for c.1726G>A and c.2065G>A in the Pompe Registry may be underestimated, reflecting the reporting physicians’ understanding that pseudodeficiency variants are not pathogenic and, therefore, they may not report them to the Registry. The same seems to be the case for c.271G>A, another common pseudodeficiency variant in Pompe disease. It is typically found in Caucasian populations and leads to a decreased affinity of GAA for glycogen, but not to disease‐causing GAA deficiency (Martiniuk, Bodkin, Tzall, & Hirschhorn, 1990).

Variant c.2560C>T, the most common globally, fourth in Europe, and also first in North America and Latin America among the Group A patients, derives its high ranking from its origin in northern Africa. It was brought to North America as well as to Brazil through the slave trade (Becker et al., 1998; Nino et al., 2013), explaining its high reported frequency in North America and Latin America. Homozygosity among Group A patients points to its very detrimental effect.

Despite the reported overall low frequency of c.340_341insT, this variant seems to be common in Registry patients from the Middle East region and gives rise to the classic infantile phenotype when occurring in homozygotes.

### 4.2 Limitations of the analysis

As with many types of data collection initiatives, there are associated limitations to the use of information entered into registries, including the Pompe Registry. Physicians and their teams enter data voluntarily with informed consent of patients. However, as noted, healthcare teams at sites may not enter data into the Registry the same or consistently or with the same level of detail or accuracy in regard to information about the details of *GAA* sequence analysis. Patient privacy has to be respected and guaranteed and in accordance with the informed consent provided by patients. As a result, only 53 of the 80 identified unique novel variants were reported for patients who had provided updated informed consent to allow the data to be submitted to a public database. Despite these limitations, we believe that the distribution and sharing of information contained in registries overall, and in the Pompe Registry specifically, can contribute substantially to the understanding of rare conditions like Pompe disease.

The study reported here does not provide the *GAA* genotype for all patients, that is, the complete genotype–phenotype correlation, which was not within the scope of this analysis. Such an analysis also would not have provided the breadth of important data and information shared here. However, we did analyze the genotype–phenotype correlation in cases of homozygosity and propose that full genotype–phenotype analyses be a focus of future studies as more variant data are added to the Registry.

Admittedly, age may not be an ideal criterion for classification of patients because Pompe disease is a clinical spectrum and results can potentially be biased. Within the spectrum, severity is markedly important for phenotypic characteristic of the disease. Only the most severe phenotype can be clearly distinguished by enzyme activity lower than 1% and cardiomyopathy before 12 months of age. Above that threshold, the spectrum is much harder to be further subdivided according to signs and symptoms and age due to differences in assessments and clinical practice. Furthermore, correlations to enzyme activity and other measurable parameters is also challenging across the spectrum. However, classifying patients by age can still be useful when investigating the association between variants and phenotypes as they also depend on patient ages.

We acknowledge that in some instances, other reported data may seem contradictory to what is known. This is due in part to the nature of reporting results from observational data. For example, although CRIM determination is only relevant for Group A, <5 patients in Group B were classified as CRIM‐negative based on information entered on the Registry CRF by the reporting site (Table 1). Determination of CRIM status is important because CRIM‐negative patients completely lack endogenous GAA enzyme and tend to respond less favorably to enzyme replacement therapy (ERT) and/or develop higher antibody titers while receiving ERT (Banugaria et al., 2011; Kishnani et al., 2010). By definition, patients in Groups B and C should only be CRIM‐positive. However, as noted previously, we do not know how CRIM status was determined and entered into the Registry and can only provide the data as reported. In addition, the homozygous substitution (missense) variants c.1843G>A, c.1933G>A, and c.1935C>A, typically found in classic infantile Pompe disease patients, were reported in both Groups A and B (Table 6). Upon careful review, the patients in Group B appear to have had cardiomyopathy reported in the Registry after 12 months of age and are expected to be in Group A. The homozygous deletion (large) variant c.2481+102_2646+31del, also typically found in classic infantile Pompe disease, was reported in Group A as well as in Group C (Table 6). This may reflect that information was reported incorrectly for the patients in Group C or may be the result of a technical error in the genetic analysis.

Overall, we believe the number of misclassified patients is low. Alternatively, unexpected results (i.e., variants reported in patients not typically associated with a phenotype) may be correct and suggest that other variants may contribute to a patient's disease severity and manifestations.

### 4.3 Data from the Pompe Registry and future directions

In summary, data reported in the Pompe Registry provide insight in the distribution of *GAA* variants across the globe and across the clinical spectrum, add to the number and diversity of *GAA* variants registered in public databases through published data sharing, provide a first indication of the severity degree of these variants, and assist in diagnostic practice and outcome prediction. Future analyses of variant data from the Pompe Registry from larger patient populations will add to our knowledge and understanding.

## CONFLICT OF INTERESTS

The Pompe Registry is sponsored by Sanofi Genzyme, Cambridge, MA.

Arnold J.J. Reuser (AJJR) has no conflicts of interest to declare.

Ans T. van der Ploeg (AvdP) has received consulting fees from Sanofi Genzyme and has provided consulting services, participated in advisory boards meetings and received grants for premarketing studies and research from industries via agreements between Erasmus MC and the industry.

Yin‐Hsiu Chien (Y‐HC) has received research support, consulting fees, reimbursement for attending symposium and other expenses, and fees for non‐CME/CE Services from Sanofi Genzyme.

Juan Llerena, Jr., (JL) is a member of the Pompe Registry International Advisory Board and has received reimbursement for participation as a speaker at lectures and symposia participation from Sanofi Genzyme.

Mary‐Alice Abbott (M‐AA) has received reimbursement for expenses related to attending Pompe Registry meetings from Sanofi Genzyme and compensation for attendance at meetings of the Scientific Advisory Board NA Pompe Registry from Sanofi Genzyme.

Paula R. Clemens (PRC) has received research funding from Sanofi, the NIH, MDA, NS Pharma, and Amicus Therapeutics; reimbursement for attending symposium and other expenses from Sanofi, MDA, NS Pharma, and Amicus Therapeutics; and consulting fees from UCB Biopharma, Spark Therapeutics, and Pfizer.

Virginia E. Kimonis (VEK) has received research support from Sanofi Genzyme for the Pompe Exercise project and participates in the Pompe Registry which is funded by Sanofi Genzyme.

Nancy Leslie (NL) has received honoraria for participation in the Pompe Registry Advisory Board from Sanofi Genzyme.

Roberto Araujo (RA) and Magali Periquet (MP) are employees of Sanofi Genzyme. RA and MP also own shares of Sanofi Genzyme.

Sonia S. Maruti was an employee of Sanofi Genzyme at the time of this analysis. Her current affiliation is Boehringer Ingelheim.

Bernd‐Jan Sanson (BJS) was an employee of Sanofi Genzyme at the time of this analysis. His current affiliation is Kiadis Pharma, Amsterdam, The Netherlands.

Antonio Toscano (AT) has received reimbursement for participation either as a speaker for lectures and symposia or as a Pompe Registry board member from Sanofi Genzyme.

Priya S. Kishnani (PSK) has received consulting fees, honoraria, and/or research funding from Sanofi Genzyme, Amicus Therapeutics, Baebies, Shire Pharmaceuticals, Alexion and the Lysosomal Disease Network, and is a member of the Pompe and Gaucher Disease Registry

## Supporting information

Supplementary information
