# Supplementary material for: GAA variants and phenotypes among 1,079 patients with Pompe disease: Data from the Pompe Registry
Source: Hum Mutat. 2019 Aug 7;40(11):2146–64. doi: 10.1002/humu.23878 (PMC6852536; doi:10.1002/humu.23878)
Supplement: Supplementary file 1 — Supplementary information [file HUMU-40-2146-s001.pdf]

SUPPORTING INFORMATION

Description of Methods Used to Identify Novel (Those Not Previously Published or Identified) Variants and the Likely Impact of These Novel Variants

Allele frequencies used to identify novel variants were extracted from the following sources, with hyperlinks to their online websites and references with details and descriptions of each:

- The Genome Aggregation Database (http://gnomad.broadinstitute.org/) (Genome Aggregation Database (gnomAD), 2017)
- The Exome Aggregation Consortium (http://exac.broadinstitute.org/) (Lek et al., 2016)
- The NHLBI Exome Sequencing Project (https://esp.gs.washington.edu/drupal/) (Tennessen et al., 2012)
- 1000 genome project consortium (http://www.internationalgenome.org/) (Genomes Project Consortium et al., 2015)
- National Center for Biotechnology Information Database Resources (NCBI Resource Coordinators, 2014)

The relationship between variants and human phenotype was analyzed using the public archive Clinvar (Landrum et al., 2014), and the Human Genome Mutation Database (HGMD) (Stenson et al., 2014).

The likely impact of variant was predicted using bioinformatics algorithms, such as PROVEAN (Choi et al., 2012), SIFT (Sim et al., 2012), PolyPhen (Adzhubei et al., 2010), and FATHMM (Shihab et al., 2015), as described below, and based on a number of criteria recommended in the ACMG Standards and Guidelines for the interpretation of sequence variants (Richards et al., 2015) and Guidelines for Reporting and Using Prediction Tools for Genetic Variant Analysis (Vihinen et al., 2012). Results are provided in TABLE 3 of the main manuscript.

Combination of potentially multiple records in dbNSFP when transcripts with different Ref and Alt amino acids overlapped at a specific position was performed. The worst of the predictions was kept for SIFT, PolyPhen, and FATHMM. If SIFT was smaller than 0.05 (rankscore>0.55) the corresponding NS was predicted as "Damaging"; otherwise it was predicted as "Tolerated". PolyPhen-2 prediction based on HumVar, was "probably damaging", HVAR score in [0.909,1] or rankscore in [0.62955,0.9711]); "possibly damaging", HVAR in [0.447,0.908] or rankscore in [0.44359,0.62885]); and "benign", HVAR score in [0,0.446] or rankscore in [0.01281,0.44315]). Score cutoff for binary classification was 0.5 for HVAR score or 0.45998 for rankscore, i.e., the prediction was "neutral" if the HVAR score was smaller than 0.5 (rankscore smaller than 0.45998), and "deleterious" if the HVAR score was larger than 0.5 (rankscore larger than 0.45998). If a FATHMM-MKL_coding_score was >0.5 (or rankscore >0.28317) the corresponding nsSNV was predicted as "Damaging"; otherwise it was predicted as "Tolerated".

SUPPORTING REFERENCES

Adzhubei, I. A., Schmidt, S., Peshkin, L., Ramensky, V. E., Gerasimova, A., Bork, P., . . . Sunyaev, S. R. (2010). A method and server for predicting damaging missense mutations. *Nature Methods, 7*(4), 248-249. doi: 10.1038/nmeth0410-248

Choi, Y., Sims, G. E., Murphy, S., Miller, J. R., & Chan, A. P. (2012). Predicting the functional effect of amino acid substitutions and indels. *PLoS One, 7*(10), e46688. doi: 10.1371/journal.pone.0046688

Genome Aggregation Database (gnomAD). (2017). GAA glucosidase, alpha; acid. Retrieved June 30, 2017 from https://gnomad.broadinstitute.org/gene/ENSG00000171298.

Genomes Project Consortium, Auton, A., Brooks, L. D., Durbin, R. M., Garrison, E. P., Kang, H. M., . . . Abecasis, G. R. (2015). A global reference for human genetic variation. *Nature, 526*(7571), 68-74. doi: 10.1038/nature15393

Landrum, M. J., Lee, J. M., Riley, G. R., Jang, W., Rubinstein, W. S., Church, D. M., & Maglott, D. R. (2014). ClinVar: public archive of relationships among sequence variation and human phenotype. *Nucleic Acids Research, 42*(Database issue), D980-D985. doi: 10.1093/nar/gkt1113

Lek, M., Karczewski, K. J., Minikel, E. V., Samocha, K. E., Banks, E., Fennell, T., . . . Exome Aggregation, C. (2016). Analysis of protein-coding genetic variation in 60,706 humans. *Nature, 536*(7616), 285-291. doi: 10.1038/nature19057

NCBI Resource Coordinators. (2014). Database Resources for the National Center for Biotechnology Information. *Nucleic Acids Research, 42*(Database Issue), D7-D17.

Richards, S., Aziz, N., Bale, S., Bick, D., Das, S., Gastier-Foster, J., . . . Committee, A. L. Q. A. (2015). Standards and guidelines for the interpretation of sequence variants: a joint consensus recommendation of the American College of Medical Genetics and Genomics and the Association for Molecular Pathology. *Genetics in Medicine, 17*(5), 405-424. doi: 10.1038/gim.2015.30

Shihab, H. A., Rogers, M. F., Gough, J., Mort, M., Cooper, D. N., Day, I. N., . . . Campbell, C. (2015). An integrative approach to predicting the functional effects of non-coding and coding sequence variation. *Bioinformatics, 31*(10), 1536-1543. doi: 10.1093/bioinformatics/btv009

Sim, N. L., Kumar, P., Hu, J., Henikoff, S., Schneider, G., & Ng, P. C. (2012). SIFT web server: predicting effects of amino acid substitutions on proteins. *Nucleic Acids Research, 40*(Web Server issue), W452-457. doi: 10.1093/nar/gks539

Stenson, P. D., Mort, M., Ball, E. V., Shaw, K., Phillips, A., & Cooper, D. N. (2014). The Human Gene Mutation Database: building a comprehensive mutation repository for clinical and molecular genetics, diagnostic testing and personalized genomic medicine. *Human Genetics, 133*(1), 1-9. doi: 10.1007/s00439-013-1358-4

Tennessen, J. A., Bigham, A. W., O'Connor, T. D., Fu, W., Kenny, E. E., Gravel, S., . . . Project, N. E. S. (2012). Evolution and functional impact of rare coding variation from deep sequencing of human exomes. *Science, 337*(6090), 64-69. doi: 10.1126/science.1219240

Vihinen, M., den Dunnen, J. T., Dalgleish, R., & Cotton, R. G. (2012). Guidelines for establishing locus specific databases. *Human Mutation, 33*(2), 298-305. doi: 10.1002/humu.21646

SUPPORTING INFORMATION TABLE S1. Distribution of Unique Known Variants Among Patients in Phenotypic Subgroups[†]

Location	DNA[‡]	Protein[§]	N Variants	Group[¶]
intron1	c.-32-13T>G	p.?	689	B,C
intron1	c.-32-17_-32-10delinsTCCCTGCTGAGCCTCCTACAGGCCTCCCG	p.?	<5	A,B
intron1	c.-32-3C>A	p.?	<5	C
intron1	c.-32-3C>G	p.?	<5	C
exon2	c.3G>A	p.(Met1?)	<5	C
exon2	c.40_47del	p.(Ala14ArgfsTer18)	<5	A
exon2	c.118C>T	p.(Arg40Ter)	8	B,C
exon2	c.236_246del	p.(Pro79ArgfsTer12)	<5	A,B
exon2	c.241C>T	p.(Gln81Ter)	<5	B,C
exon2	c.258C>A	p.(Pro86Pro)	<5	C
exon2	c.258dup	p.(Asn87fsTer9)	13	A,B,C
exon2	c.265C>T	p.(Arg89Cys)	<5	C
exon2	c.266G>A	p.(Arg89His)	<5	A
exon2	c.271G>A	p.(Asp91Asn)	<5	C
exon2	c.271del	p.(Asp91fsTer51)	<5	C
exon2	c.307T>C	p.(Cys103Arg)	<5	C
exon2	c.307T>G	p.(Cys103Gly)	34	A,B,C
exon2	c.323G>A	p.(Cys108Ser)	<5	B
exon2	c.340_341insT	p.(Lys114fsTer32)	6	A
exon2	c.352C>T	p.(Gln118Ter)	<5	A
exon2	c.364A>G	p.(Met122Val)	<5	B,C
exon2	c.365del	p.(Met122ArgfsTer20)	<5	C
exon2	c.378_379del	p.(Cys127LeufsTer18)	<5	A,C
exon2	c.379_380del	p.(Cys127LeufsTer18)	<5	C
exon2	c.380G>A	p.(Cys127Tyr)	<5	C
exon2	c.424_440del	p.(Ser142LleufsTer29)	<5	B
exon2	c.461_469del	p.(Arg154_Thr156del)	<5	C
exon2	c.482_483del	p.(Pro161fsTer15)	7	C
exon2	c.502C>T	p.(Arg168Trp)	<5	B
exon2	c.510C>T	p.(Asp170Asp)	<5	B,C
exon2	c.525_526del	p.(Asn177ProfsTer11)	5	A,C
exon2	c.525del	p.(Glu176ArgfsTer45)	111	A,B,C
exon2	c.546G>A	p.?	9	B,C
intron2	c.546+1G>T	p.?	<5	C
intron2	c.546+2_5del	p.?	<5	A
exon3	c.568C>T	p.(Arg190Cys)	<5	B
exon3	c.569G>A	p.(Arg190His)	<5	B,C
exon3	c.572A>G	p.(Tyr191Cys)	<5	A,B
exon3	c.573C>A	p.(Tyr191Ter)	<5	B,C
exon3	c.655G>A	p.(Gly219Arg)	14	A,B,C
exon3	c.670C>T	p.(Arg224Trp)	16	A,B,C
exon3	c.691C>T	p.(Leu231Leu)	<5	B

Location	DNA[‡]	Protein[§]	N Variants	Group[¶]
intron3	c.692+1G>C	p.?	<5	C
intron3	c.692+5G>T	p.?	7	B,C
intron3	c.693-1G>C	p.?	<5	C
exon4	c.701C>A	p.(Thr234Lys)	<5	B,C
exon4	c.716del	p.(Leu239fsTer29)	<5	A
exon4	c.722_723del	p.(Phe241CysfsTer88)	<5	A
exon4	c.742del	p.(Leu248ProfsTer20)	<5	C
exon4	c.743T>C	p.(Leu248Pro)	<5	C
exon4	c.752C>T	p.(Ser251Leu)	<5	B
exon4	c.761C>T	p.(Ser254Leu)	<5	B
exon4	c.784G>A	p.(Glu262Lys)	12	A,B,C
exon4	c.794del	p.(Ser265IlefsTer3)	<5	A,C
exon4	c.811A>G	p.(Thr271Ala)	<5	B
exon4	c.827_845del	p.(Ile276ThrfsTer32)	<5	C
exon4	c.836G>A	p.(Trp279Ter)	<5	C
exon4	c.841C>T	p.(Arg281Trp)	<5	B
exon4	c.853C>T	p.(Pro285Ser)	<5	C
exon4	c.854C>G	p.(Pro285Arg)	<5	A,C
exon5	c.868A>G	p.(Asn290Asp)	<5	C
exon5	c.871C>T	p.(Leu291Phe)	<5	A,B
exon5	c.872T>C	p.(Leu291Pro)	<5	A
exon5	c.875A>G	p.(Tyr292Cys)	<5	B
exon5	c.877G>A	p.(Gly293Arg)	10	A,B,C
exon5	c.883C>A	p.(His295Asn)	<5	C
exon5	c.896T>C	p.(Leu299Pro)	<5	B,C
exon5	c.923A>C	p.(His308Pro)	<5	B,C
exon5	c.925G>A	p.(Gly309Arg)	16	A,B,C
exon5	c.947A>G	p.(Asn316Ser)	<5	A
exon5	c.953T>A	p.(Met318Lys)	<5	B
exon5	c.953T>C	p.(Met318Thr)	<5	A
exon6	c.989G>A	p.(Trp330Ter)	<5	C
exon6	c.1000G>T	p.(Gly334Cys)	<5	A
exon6	c.1003G>A	p.(Gly335Arg)	<5	B
exon6	c.1040C>G	p.(Pro347Arg)	<5	C
exon6	c.1047del	p.(Ser349ArgfsTer43)	<5	B,C
exon6	c.1051del	p.(Val351CysfsTer41)	6	B,C
exon6	c.1062C>G	p.(Tyr354Ter)	<5	A,B
exon6	c.1064T>C	p.(Leu355Pro)	8	A,B,C
exon6	c.1075G>A	p.(Gly359Arg)	<5	C
intron6	c.1075+13C>T	p.?	<5	A,B
intron6	c.1076-1G>A	p.?	<5	C
intron6	c.1076-1G>C	p.?	<5	C
intron6	c.1076-22T>G	p.(Asp319_Val358delins)	6	B,C
intron6	c.1076-2A>G	p.?	<5	C
exon7	c.1082C>T	p.(Pro361Leu)	9	A,B,C
exon7	c.1099T>C	p.(Trp367Arg)	<5	A,B
exon7	c.1100G>A	p.(Trp367Ter)	<5	B
exon7	c.1106T>A	p.(Leu369Gln)	<5	C

Location	DNA[‡]	Protein[§]	N Variants	Group[¶]
exon7	c.1115A>T	p.(His372Leu)	5	A,C
exon7	c.1124G>A	p.(Arg375His)	<5	C
exon7	c.1124G>T	p.(Arg375Leu)	<5	C
exon7	c.1128_1129delinsC	p.(Trp376CysfsTer16)	6	B,C
exon7	c.1129G>C	p.(Gly377Arg)	<5	A
exon7	c.1134C>G	p.(Tyr378Ter)	<5	C
exon7	c.1143del	p.(Ala382LeufsTer10)	6	C
exon7	c.1157dup	p.(Val387GlyfsTer119)	<5	A
exon7	c.1190C>T	p.(Pro397Leu)	<5	A,B,C
exon7	c.1193del	p.(Leu398ArgfsTer42)	<5	C
intron7	c.1194+2T>A	p.?	<5	C
intron7	c.1195-2A>G	p.?	<5	A
intron7	c.1195-8G>A	p.?	<5	C
exon8	c.1197_1208del	p.(Val400_Asn403del)	<5	A
exon8	c.1210G>A	p.(Asp404Asn)	13	A,B,C
exon8	c.1211A>G	p.(Asp404Gly)	<5	A,C
exon8	c.1219T>C	p.(Tyr407His)	<5	C
exon8	c.1221C>A	p.(Tyr407Ter)	<5	A
exon8	c.1222A>G	p.(Met408Val)	<5	C
exon8	c.1239C>G	p.(Asp413Glu)	<5	C
exon8	c.1281G>T	p.(Met427Ile)	<5	A
exon8	c.1286A>G	p.(Gln429Arg)	<5	A
exon8	c.1291_1299del	p.(Leu431_Gln433del)	<5	C
exon8	c.1293_1312del	p.(Gln433AspfsTer66)	<5	B
exon8	c.1309C>T	p.(Arg437Cys)	<5	C
exon8	c.1316T>A	p.(Met439Lys)	<5	C
exon8	c.1320_1322del	p.(Met440del)	<5	B
exon8-15	c.1195-18_2190-20del	p.(Asp399ValfsTer6)	<5	A
intron8	c.1326+1G>A	p.?	<5	C
intron8	c.1327-2A>G	p.?	<5	A
exon9	c.1333G>C	p.(Ala445Pro)	<5	B
exon9	c.1354_1372del	p.(Ala452_Pro458del_458fs Ter19)	<5	C
exon9	c.1355del	p.(Ser454AlafsTer23)	<5	C
exon9	c.1370C>T	p.(Pro457Leu)	<5	B
exon9	c.1373_1375del	p.(Asp459del)	<5	C
exon9	c.1375G>A	p.(Asp459Asn)	<5	C
exon9	c.1396G>T	p.(Val466Phe)	<5	A,C
exon9	c.1396del	p.(Val466PhefsTer11)	<5	A,B,C
exon9	c.1402A>T	p.(Ile468Phe)	5	A,B,C
exon9	c.1408_1410del	p.(Asn470del)	<5	A,B
exon9	c.1411_1414del	p.(Glu471ProfsTer5)	<5	A
exon9	c.1437G>A	p.(Asp443_Lys479del)	6	B,C
intron9	c.1437+1G>A	p.?	<5	A
intron9	c.1437+2T>C	p.(Asp443_Lys479del)	<5	A,B,C
intron9	c.1438-1G>C	p.?	<5	A,B,C
intron9	c.1438-1G>T	p.?	<5	C
intron9	c.1438-2A>G	p.?	<5	B

Location	DNA[‡]	Protein[§]	N Variants	Group[¶]
exon10	c.1441T>C	p.(Trp481Arg)	7	A,B,C
exon10	c.1441del	p.(Trp481GlyfsTer39)	<5	A
exon10	c.1445C>G	p.(Pro482Arg)	<5	C
exon10	c.1445C>T	p.(Pro482Leu)	<5	B
exon10	c.1446del	p.(Ser484ProfsTer36)	<5	C
exon10	c.1447G>A	p.(Gly483Arg)	6	A,B,C
exon10	c.1456G>C	p.(Ala486Pro)	<5	C
exon10	c.1456G>T	p.(Ala486Ser)	<5	C
exon10	c.1465G>A	p.(Asp489Asn)	13	A,B,C
exon10	c.1466A>G	p.(Asp489Gly)	<5	A
exon10	c.1478C>T	p.(Pro493Leu)	6	C
exon10	c.1495T>A	p.(Trp499Arg)	<5	B
exon10	c.1496G>A	p.(Trp499Ter)	<5	A
exon10	c.1498_1512del	p.(Asp501_Glu505del)	<5	C
exon10	c.1537G>A	p.(Asp513Asn)	<5	B
exon10	c.1548G>A	p.(Trp516Ter)	17	A,B,C
intron10	c.1551+1G>A	p.(Val480_Ile517del)	<5	C
intron10	c.1551+1G>C	p.(Val480_Ile517del)	7	C
intron10	c.1551+1G>T	p.(Val480_Ile517del)	<5	A
intron10	c.1551+3_1551+6del	p.(Val480_Ile517del)	<5	C
intron10	c.1551+49A>C	p.?	<5	C
exon11	c.1556T>C	p.(Met519Thr)	<5	B
exon11	c.1560C>G	p.(Asn520Lys)	<5	C
exon11	c.1561G>A	p.(Glu521Lys)	8	A,B,C
exon11	c.1564C>A	p.(Pro522Thr)	<5	B
exon11	c.1564C>G	p.(Pro522Ala)	9	A,B,C
exon11	c.1564C>T	p.(Pro522Ser)	<5	A
exon11	c.1568C>A	p.(Ser523Tyr)	<5	B
exon11	c.1581A>G	p.(Arg527Arg)	<5	C
exon11	c.1583G>C	p.(Gly528Ala)	<5	B
exon11	c.1594G>A	p.(Gly532Ser)	<5	C
exon11	c.1610del	p.(Glu537GlyfsTer41)	<5	B,C
exon11	c.1634C>T	p.(Pro545Leu)	7	B,C
exon11	c.1636G>C	p.(Gly546Ala)	<5	B
intron11	c.1636+1G>C	p.?	5	B,C
intron11	c.1637-2A>G	p.?	<5	A
exon12	c.1642G>T	p.(Val548Phe)	<5	A,C
exon12	c.1645G>C	p.(Gly549Arg)	<5	B
exon12	c.1650dup	p.(Thr551AspfsTer85)	<5	A
exon12	c.1654del	p.(Leu552SerfsTer26)	<5	A
exon12	c.1655T>C	p.(Leu552Pro)	19	A,B,C
exon12	c.1669A>T	p.(Ile557Phe)	<5	B,C
exon12	c.1694_1697del	p.(Leu565ProfsTer12)	<5	C
exon12	c.1703A>T	p.(His568Leu)	<5	A,C
exon12	c.1705dup	p.(Tyr569LeufsTer67)	<5	A
exon12	c.1710C>G	p.(Asn570Lys)	<5	B,C
exon12	c.1716C>G	p.(His572Gln)	<5	B
exon12	c.1717A>C	p.(Asn573His)	<5	C

Location	DNA[‡]	Protein[§]	N Variants	Group[¶]
exon12	c.1724A>C	p.(Tyr575Ser)	<5	A
exon12	c.1726G>A	p.(Gly576Ser)	17	A,B,C
exon12	c.1735G>A	p.(Glu579Lys)	<5	A,B,C
exon12	c.1748C>T	p.(Ser583Phe)	<5	C
exon12	c.1754G>A	p.(Arg585Lys)	<5	C
intron12	c.1754+1G>A	p.?	<5	A,C
intron12	c.1754+2T>A	p.?	<5	A
intron12	c.1754+2T>C	p.?	<5	B
exon13	c.1781G>A	p.(Arg594His)	<5	B,C
exon13	c.1781G>C	p.(Arg594Pro)	<5	B
exon13	c.1796C>A	p.(Ser599Tyr)	<5	A,B,C
exon13	c.1798C>T	p.(Arg600Cys)	5	A,B,C
exon13	c.1799G>A	p.(Arg600His)	13	A,B,C
exon13	c.1802C>G	p.(Ser601Trp)	<5	A,C
exon13	c.1802C>T	p.(Ser601Leu)	<5	A,C
exon13	c.1804A>G	p.(Thr602Ala)	<5	B
exon13	c.1819_1836del	p.(Gly607_His612del)	<5	B,C
exon13	c.1822C>T	p.(Arg608Ter)	7	A,B,C
exon13	c.1826dup	p.(Tyr609Ter)	<5	C
exon13	c.1827del	p.(Tyr609Ter)	<5	B,C
exon13	c.1832G>A	p.(Gly611Asp)	<5	C
exon13	c.1835A>G	p.(His612Arg)	<5	B
exon13	c.1841C>A	p.(Thr614Lys)	7	A,B,C
exon13	c.1843G>A	p.(Gly615Arg)	9	A,B,C
exon13	c.1844G>A	p.(Gly615Glu)	5	A,B
exon13	c.1844_1846del	p.(Gly615del)	<5	C
exon13	c.1846G>A	p.(Asp616Asn)	<5	A
exon13	c.1856G>A	p.(Ser619Asn)	<5	B
exon13	c.1857C>G	p.(Ser619Arg)	<5	B,C
exon13	c.1880C>T	p.(Ser627Phe)	5	A,C
intron13	c.1888+1G>A	p.?	<5	B,C
exon14	c.1903A>G	p.(Asn635Asp)	<5	B
exon14	c.1912G>T	p.(Gly638Trp)	8	A,B,C
exon14	c.1913G>A	p.(Gly638Glu)	<5	A
exon14	c.1913G>T	p.(Gly638Val)	<5	B
exon14	c.1923G>A	p.(Leu641Leu)	<5	C
exon14	c.1924G>T	p.(Val642Phe)	<5	B
exon14	c.1927G>A	p.(Gly643Arg)	21	A,B,C
exon14	c.1933G>A	p.(Asp645Asn)	14	A,B,C
exon14	c.1933G>C	p.(Asp645His)	<5	A
exon14	c.1933G>T	p.(Asp645Tyr)	<5	A,C
exon14	c.1935C>A	p.(Asp645Glu)	42	A,B,C
exon14	c.1941C>G	p.(Cys647Trp)	<5	A,C
exon14	c.1942G>A	p.(Gly648Ser)	7	A,C
exon14	c.1951_1952delinsT	p.(Gly651SerfsTer45)	<5	C
exon14	c.1962_1964del	p.(Glu656del)	<5	A,C
exon14	c.1978C>T	p.(Arg660Cys)	<5	B
exon14	c.1979G>A	p.(Arg660His)	9	A,B,C

Location	DNA[‡]	Protein[§]	N Variants	Group[¶]
exon14	c.1993G>A	p.(Gly665Arg)	<5	B
exon14	c.2014C>T	p.(Arg672Trp)	7	B,C
exon14	c.2015G>A	p.(Arg672Gln)	5	A,B,C
exon14	c.2023_2025del	p.(Ans675del)	<5	A
exon14	c.2024_2026del	p.(Asn675del)	<5	A
exon14	c.2040G>A	p.?	<5	A
intron14	c.2041-1G>A	p.?	<5	C
exon15	c.2051C>T	p.(Pro684Leu)	<5	A
exon15	c.2055C>A	p.(Tyr685Ter)	<5	C
exon15	c.2066_2070dup	p.(Ala691fsTer6)	<5	C
exon15	c.2078dup	p.(Ala694GlnfsTer42)	<5	A
exon15	c.2104C>T	p.(Arg702Cys)	<5	A,B,C
exon15	c.2105G>A	p.(Arg702His)	<5	B
exon15	c.2105G>T	p.(Arg702Leu)	<5	A,C
exon15	c.2132C>G	p.(Thr711Arg)	<5	B,C
exon15	c.2135T>C	p.(Leu712Pro)	<5	B,C
exon15	c.2136_2137del	p.(Phe713ProfsTer23)	<5	C
exon15	c.2161G>T	p.(Glu721Ter)	<5	B
exon15	c.2173C>T	p.(Arg725Trp)	8	A,C
intron15	c.2189+1G>A	p.?	<5	B
exon16	c.2210C>A	p.(Thr737Asn)	<5	B
exon16	c.2214G>A	p.(Trp738Ter)	<5	B,C
exon16	c.2219_2220del	p.(Val740GlyfsTer55)	<5	A,C
exon16	c.2222A>T	p.(Asp741Val)	<5	C
exon16	c.2227C>T	p.(Gln743Ter)	<5	A
exon16	c.2228A>G	p.(Gln743Arg)	5	B,C
exon16	c.2236T>C	p.(Trp746Arg)	<5	A
exon16	c.2237G>A	p.(Trp746Ter)	20	A,B,C
exon16	c.2237G>C	p.(Trp746Ser)	<5	B
exon16	c.2238G>A	p.(Trp746Ter)	<5	A,B
exon16	c.2238G>C	p.(Trp746Cys)	38	B,C
exon16	c.2242G>T	p.(Glu748Ter)	<5	C
exon16	c.2242dup	p.(Glu748fsTer48)	9	B,C
exon16	c.2269C>T	p.(Gln757Ter)	<5	C
exon16	c.2274dup	p.(Gly759ArgfsTer6)	<5	A
exon16	c.2281delinsAT	p.(Ala761fs)	<5	B,C
exon16	c.2294G>A	p.(Gly765Asp)	<5	A
exon16	c.2296T>A	p.(Tyr766Asn)	<5	A
exon16	c.2297A>C	p.(Tyr766Ser)	8	A,B,C
exon16	c.2297A>G	p.(Tyr766Cys)	<5	B
exon16	c.2298_2301delinsAAAGTA	p.(Tyr766Ter)	<5	C
exon16	c.2303C>T	p.(Pro768Leu)	<5	A
exon16	c.2320G>A	p.(Asn774Asn)	<5	C
exon16	c.2322_2323insGGTGAGTCTGCAAACGGGGAGT	p.(Leu775GlyfsTer70)	<5	C
intron16	c.2331+1G>A	p.?	<5	A
intron16	c.2331+20A>G	p.?	<5	B,C
intron16	c.2331+2T>A	p.?	<5	A,B,C

Location	DNA‡	Protein§	N Variants	Group¶
exon17	c.2338A>G	p.(Ile780Val)	<5	C
exon17	c.2395C>T	p.(His799Tyr)	<5	C
exon17	c.2408_2426del	p.(Gln803ProfsTer39)	<5	A
exon17	c.2431del	p.(Leu811TrpfsTer37)	<5	B,C
exon17	c.2431dup	p.(Leu811fsTer73)	<5	B
exon17	c.2446G>A	p.(Val816Ile)	<5	B
exon17	c.2474C>G	p.(Pro825Arg)	<5	B,C
intron17	c.2481+102_2646+31del	p.(Gly828_Asn882del)	80	A,B,C
exon18	c.2495_2496del	p.(Thr832AsnfsTer51)	6	A,B
exon18	c.2501_2502del	p.(Thr834ArgfsTer49)	<5	A,B
exon18	c.2512C>T	p.(Gln838Ter)	<5	A,C
exon18	c.2528T>C	p.(Leu843Pro)	<5	A
exon18	c.2530_2541del	p.(Arg844_Leu847del)	10	B,C
exon18	c.2544del	p.(Lys849ArgfsTer39)	<5	C
exon18	c.2560C>T	p.(Arg854Ter)	45	A,B,C
exon18	c.2604del	p.(Leu868fs)	<5	C
exon18	c.2608C>T	p.(Arg870Ter)	17	A,B,C
intron18	c.2646+2T>A	p.(Val876_Asn882del)	<5	A
intron18	c.2647-20T>G	p.?	<5	C
exon19	c.2662G>T	p.(Glu888Ter)	14	A,B,C
exon19	c.2706del	p.(Lys903ArgfsTer2)	<5	C
exon19	c.2707_2709del	p.(Lys903del)	<5	A
exon19	c.2725G>A	p.(Val909Met)	<5	B
exon19	c.2738C>G	p.(Pro912Arg)	<5	B,C
exon19	c.2741delinsGAC	p.(Gln914fsTer30)	<5	A
exon19	c.2744A>C	p.(Gln915Pro)	<5	B
exon19	c.2770T>C	p.(Ser924Pro)	<5	A
exon19	c.2780C>T	p.(Thr927Ile)	<5	C
intron19	c.2799+4A>G	p.?	<5	C
exon20	c.2815_2816del	p.(Val939LeufsTer78)	5	A,B,C
exon20	c.2841_2842insT	p.(Leu948SerfsTer70)	<5	A
exon20	c.2846T>A	p.(Val949Asp)	<5	A

†The phenotypic subgroups are defined as follows. Group A: onset of symptoms ≤12 months of age with cardiomyopathy (patients classified as classic infantile Pompe disease); Group B: onset of symptoms ≤12 years of age (includes patients with onset of symptoms ≤12 months of age without cardiomyopathy and not included in Group A); Group C: onset of symptoms >12 years of age.
‡c.1726G>A is a common pseudodeficiency variant and is linked to other variants (see main text).
§The protein is provided where data were available. The value p.? is used to indicate that the impact on the RNA is unknown.
¶Designates the phenotypic subgroup(s) of patients in which a variant is reported: Group A, Group B, and/or Group C.
Note: Impact of the variants in the table is provided in the Pompe disease mutation database at http://www.pompecenter.nl (>molecular aspects>Pompe variants database).

SUPPORTING INFORMATION TABLE S2. Novel Variants (with Appropriate Consent†) from the Pompe Registry Submitted to, and Accepted by, the Single Nucleotide Polymorphism Database (dbSNP) with submission ID SUB4205047 (http://www.ncbi.nlm.nih.gov/SNP/) and the Leiden Open Variation Database (LOVD; https://www.lovd.nl/).

Variant	Protein	Genomic Location of Variant	dbSNP Submission Number	LOVD Variant ID
c.665T>G	p.(Val222Gly)	NC_000017.10:g.78079666T>G	ss2137543935	0000500691
c.692T>C	p.(Leu231Pro)	NC_000017.10:g.78079693T>C	ss3654261701	0000500692
c.692+1G>T	p.?	NC_000017.10:g.78079694G>T	ss3654261702	0000500693
c.693-2A>C	p.?	NC_000017.10:g.78081354A>C	ss3654261703	0000500694
c.766_784del	p.(Tyr256SerfsTer6)	NC_000017.10:g.78081429_78081447del	ss3654261704	0000500695
c.878G>T	p.(Gly293Val)	NC_000017.10:g.78081618G>T	ss3654261705	0000500696
c.930_932del	p.(Phe311del)	NC_000017.10:g.78081670_78081672del	ss3654261706	0000500697
c.950C>T	p.(Ala317Val)	NC_000017.10:g.78081690C>T	ss3654261707	0000500698
c.994_995insTT	p.(Ser332PhefsTer61)	NC_000017.10:g.78082127_78082128insTT	ss3654261708	0000500699
c.1109G>A	p.(Gly370Asp)	NC_000017.10:g.78082321G>A	ss3654261709	0000500700
c.1114C>G	p.(His372Asp)	NC_000017.10:g.78082326C>G	ss3654261710	0000500701
c.1114C>T	p.(His372Tyr)	NC_000017.10:g.78082326C>T	ss3654261711	0000500702
c.1121G>A	p.(Cys374Tyr)	NC_000017.10:g.78082333G>A	ss3654261712	0000500703
c.1211A>T	p.(Asp404Val)	NC_000017.10:g.78082512A>T	ss3654261713	0000500704
c.1388_1406del	p.(Arg463ProfsTer8)	NC_000017.10:g.78083805_78083823del	ss3654261714	0000500705
c.1409A>G	p.(Asn470Ser)	NC_000017.10:g.78083826A>G	ss3654261715	0000500853
c.1477C>T	p.(Pro493Ser)	NC_000017.10:g.78084565C>T	ss3654261717	0000500854
c.1526A>T	p.(Gln509Leu)	NC_000017.10:g.78084614A>T	ss3654261718	0000500855
c.1551+3A>T	p.?	NC_000017.10:g.78084642A>T	ss3654261719	0000500856
c.1559A>G	p.(Asn520Ser)	NC_000017.10:g.78084747A>G	ss3654261720	0000500857
c.1670T>G	p.(Ile557Ser)	NC_000017.10:g.78085815T>G	ss3654261721	0000500858
c.1681_1699dup	p.(Thr567LysfsTer75)	NC_000017.10:g.78085826_78085844dup1	ss3654261722	0000500859
c.1754+1dup	p.?	NC_000017.10:g.78085900dup	ss3654261723	0000500860
c.1825T>G	p.(Tyr609Asp)	NC_000017.10:g.78086447T>G	ss3654261724	0000500861
c.1839G>C	p.(Trp613Cys)	NC_000017.10:g.78086461G>C	ss3654261725	0000500863
c.1847dup	p.(Asp61GlufsTer20)	NC_000017.10:g.78086469dup	ss3654261726	0000500862
c.1876_1878del	p.(Ser627del)	NC_000017.10:g.78086498_78086500del	ss3654261727	0000500864
c.1944_1950del	p.(Phe649AlafsTer45)	NC_000017.10:g.78086730_78086736del	ss3654261728	0000500865
c.1961C>G	p.(Ser654Ter)	NC_000017.10:g.78086747C>G	ss3654261729	0000500866
c.2020C>T	p.(His674Tyr)	NC_000017.10:g.78086806C>T	ss3654261730	0000500867

Variant	Protein	Genomic Location of Variant	dbSNP Submission Number	LOVD Variant ID
c.2041-2A>G	p.?	NC_000017.10:g.78087015A>G	ss3654261731	0000500868
c.2084dup	p.(Met695IlefsTer70)	NC_000017.10:g.78087060dup	ss3654261732	0000500869
c.2096T>C	p.(Leu699Pro)	NC_000017.10:g.78087072T>C	ss3654261733	0000500870
c.2109del	p.(Tyr703Ter)	NC_000017.10:g.78087085del	ss3654261734	0000500872
c.2146G>C6/14/20	p.(Ala716Pro)	NC_000017.10:g.78087122G>C	ss3654261735	0000500873
c.2153_2156delinsACGCCG	p.(Val718AspfsTer47)	NC_000017.10:g.78087129_78087132delinsACGCCG	ss3654261736	0000500874
c.2237G>T	p.(Trp746Leu)	NC_000017.10:g.78090814G>T	ss3654261737	0000500875
c.2240G>A	p.(Gly747Glu)	NC_000017.10:g.78090817G>A	ss3654261738	0000500876
c.2258_2259insC	p.(Val755SerfsTer41)	NC_000017.10:g.78090835_78090836insC	ss3654261739	0000500877
c.2261dup	p.(Val755LysfsTer10)	NC_000017.10:g.78090838dup	ss3654261740	0000500878
c.2331+101del	p.?	NC_000017.10:g.78091009del536	ss3654261741	0000500879
c.2407C>T	p.(Gln803Ter)	NC_000017.10:g.78091474C>T	ss3654261742	0000500880
c.2459_2461del	p.(Ala820del)	NC_000017.10:g.78091526_78091528del	ss3654261743	0000500881
c.2460dup	p.(Gly821TrpfsTer63)	NC_000017.10:g.78091527dup	ss3654261744	0000500882
c.2480A>G	p.(Gln827Arg)	NC_000017.10:g.78091547A>G	ss3654261745	0000500883
c.2515C>T	p.(Gln839Ter)	NC_000017.10:g.78092025C>T	ss3654261746	0000500884
c.2619C>G	p.(Tyr873Ter)	NC_000017.10:g.78092129C>G	ss3654261747	0000500885
c.2655_2656del	p.(Val886GlufsTer2)	NC_000017.10:g.78092460_	ss3654261748	0000500886
c.2720T>C	p.(Leu907Pro)	NC_000017.10:g.78092525T>C	ss3654261749	0000500887
c.2740dup	p.(Gln914ProfsTer104)	NC_000017.10:g.78092545dup	ss3654261750	0000500888
c.2742dup	p.(Gln915AlafsTer10)	NC_000017.10:g.78092547dup	ss3654261751	0000500890
c.2757del	p.(Asn919LysfsTer24)	NC_000017.10:g.78092562del	ss3654261752	0000500891
c.2800-1G>C	p.?	NC_000017.10:g.78093070G>C	ss3654261753	0000500893

[†]Informed written patient consent is required to share patient data submitted to the Pompe Registry (see Methods of the main manuscript). Not all Registry patients agree to have their data shared. Only novel *GAA* variants for which appropriate updated patient consent was available were submitted to the Single Nucleotide Polymorphism Database (dSNP) and the Leiden Open Variation Database (LOVD)
